# Multicomponent Selective and Efficient Synthesis of Spiro[furo[2,3-*d*]pyrimidine-6,4′-pyrazole] Scaffold Under a Column Chromatography-Free Protocol at Room Temperature

**DOI:** 10.3390/molecules31162880

**Published:** 2026-08-18

**Authors:** Michail N. Elinson, Yuliya E. Ryzhkova, Varvara M. Kalashnikova, Mikhail P. Egorov

**Affiliations:** N. D. Zelinsky Institute of Organic Chemistry, Leninsky Prospect 47, 119991 Moscow, Russiap.varvara2001@gmail.com (V.M.K.); mpe@ioc.ac.ru (M.P.E.)

**Keywords:** multicomponent reaction, Knoevenagel–Michael reaction, cyclization, aldehyde, *N*,*N*′-dimethylbarbituric acid, 3-methyl-1-phenyl-2-pyrazolin-5-one

## Abstract

This new multicomponent tandem Knoevenagel–Michael reaction with further cyclization by the action of *N*-bromosuccinimide was discovered. The assembly of arylaldehydes, *N*,*N*′-dialkylbarbituric acids and 3-methyl-1-phenyl-2-pyrazolin-5-one by the action of NaOAc, and then oxidation of the Knoevenagel–Michael adduct by the *N*-bromosuccinimide in ethanol without any other additives at ambient temperature, resulted in the selective and efficient formation of the medicinally relevant unsymmetrical 2*H*-spiro[furo[2,3-*d*]pyrimidine-6,4′-pyrazole]-2,3′,4(2′*H*,3*H*)-trione scaffold with three pharmacology active heterocyclic rings in 73–90% yields. This multicomponent reaction is a simple and efficient route to substituted 2*H*-spiro[furo[2,3-*d*]pyrimidine-6,4′-pyrazole]-2,3′,4(2′*H*,3*H*)-triones. This multicomponent method does not require heating, long reaction times, or complex equipment. It is easy to carry out, and the procedure for isolating the final compounds is very simple. Thus, this new multicomponent method is applicable for large-scale processes. All these advantages make this method valuable for the synthesis of new potential biologically active compounds.

## 1. Introduction

Synthesizing targeted complex organic compounds with more carbon atoms in their skeletal system than in the parent organic molecules requires strategic use of various C–C bond-forming reactions (e.g., Aldol, Benzoin, Diels–Alder, Knoevenagel, Michael addition, or Suzuki–Miyaura). The tandem Knoevenagel–Michael reaction is one of the most important strategies for the formation of the new C–C bonds. It has proven to be quite versatile, and it has been widely used to generate valuable useful building blocks in organic synthesis [1]. Achievements in this area are mainly focused on the development of new catalysts and on the establishment of activation modes with simple substrates [2]. Much effort has been devoted to the target- and diversity-oriented synthesis, in which many approaches to the concise synthesis of unique and complicated scaffolds have been attempted [3].

The concept of developing reaction strategies at room temperature and normal pressure is now an emerging field of research in organic chemistry, and it is progressing steadily as a part of the recent research works directly related to green chemistry practice, leading to C–C bond-forming reactions of topical significance [4].

Multicomponent reactions (MCRs) provide a number of valuable conceptual and synthetic advantages over stepwise sequential approaches towards complex and valuable molecules. Dissimilar to conventional reactions, multistep processes are characterized by higher yields, atom economy, shorter reaction times, ecologically benign conditions, and lower costs [5]. MCRs are nowadays the main approach used in diversity-oriented synthesis with maximum structural complexity in minimum steps [6]. Unlike usual traditional methods, MCRs increase the accessible chemical space exponentially with each additional reaction organic molecule [7]. Thus, now MCRs are widely recognized for their impact on drug discovery and strongly endorsed by industry in addition to scientific research.

The multicomponent synthesis of complex heterocyclic compounds is one of the main goals in modern organic chemistry, because of their important roles in medicine, materials science, and biology. This is due to the fact that heterocycles, which have at least two different elements in a ring, form the main part of most modern drugs and have diverse applications.

The discovery of new types of selective and efficient MCRs is the main area at the forefront of modern organic synthesis. The inherent difficulty lies in controlling the selectivity and compatibility of the starting compounds [8]. Thus, the ideal method may be the simple mixing of only the participating compounds without heating to achieve the desired result in one step [9].

Privileged structures or scaffolds are versatile molecular frameworks capable of providing high-affinity ligands for diverse biological targets through functional group modification. There are used to accelerate drug discovery by providing a common starting point for designing potent and selective ligands for various diseases, such as antiviral agents, central nervous system drugs, and kinase inhibitors [10].

Barbiturates (pyrimidine-2,4,6-triones) are used in medicinal chemistry for developing anticonvulsant, sedative, and antitumor agents, owing to their diverse pharmacological activities [11]. They also serve as vital synthetic building blocks, including in supramolecular chemistry for host–guest architectures and functionalized materials [12].

Functionalized 2-pyrazolin-5-ones are useful five-membered heterocyclic pharmacophores with diverse medicinal properties, including anti-inflammatory, antimicrobial, antiviral, and anticancer activities [13]. These compounds are key scaffolds in drug development, often serving as non-steroidal anti-inflammatory agents, analgesic, or antioxidant molecules due to their ability to inhibit enzymes [13]. Antipyrine or phenazone is a well-known synthetic non-steroidal compound with efficient analgesic properties [14]. Following the synthesis of antipyrine, pyrazolone derivatives have garnered significant attention due to their potent analgesic, anti-inflammatory, and antipyretic properties, often acting as effective COX inhibitors [15]. These compounds are part of the non-steroidal anti-inflammatory drug (NSAID) class, frequently utilized for treating joint and musculoskeletal disorders [15].

Spirocycles are highly valued in medicinal chemistry for providing a unique, three-dimensional, and rigid yet adaptable framework, increasing potency and selectivity while improving drug-like properties such as solubility, metabolic stability, and pharmacokinetics compared to flatter aromatic systems [16]. The *sp*^3^-rich character of spirocycles and the resulting extended range of exit vectors spanning across all three spatial dimensions represent an exciting opportunity for expanding molecular complexity, while maintaining structural rigidity. These structures often enhance binding to biological targets by allowing for specific, fixed, and orthogonal orientations of molecular components [17]. Spiro-heterocycles garner more fascination due to their unique conformational features and vast biological applications than simple heterocycles [18].

Spirobarbiturates are versatile spiroheterocyclic compounds with significant pharmacological applications, acting as anticonvulsants and sedatives [19], hypnotic agents and CNS depressants [20], and anticancer remedies [21].

Spiropyrazolone scaffolds are also present in many medicinally relevant compounds with potent biological activities such as antitumor [22], analgesic, and anti-inflammatory activities [23]. For this reason, substantial research efforts have been invested toward the synthesis of spirocyclic pyrazolone frameworks over the last years [24].

A convenient, efficient, modern multicomponent strategy for synthesizing pharmacologically active spiroheterocyclic scaffolds involves the one-pot assembly of carbonyl compounds and different heterocyclic C-H acids. Considering our experience in multicomponent reactions with the formation of complex heterocyclic compounds [25] and biomedical applications of spiroheterocyclic scaffolds [26], we were prompted to design a convenient and efficient multicomponent strategy with the following cyclization for the efficient assembling of aldehydes, *N*,*N*′-dimethylbarbituric acid, and 3-methyl-1-phenyl-2-pyrazolin-5-one into a spirotetracyclic scaffold with three pharmacologically active heterocyclic rings.

## 2. Results and Discussion

Thus, now we would like to report our data on the selective and efficient cascade assembling of arylaldehydes **1a**–**h**, *N*,*N*′-dimethylbarbituric acid, and 3-methyl-1-phenyl-2-pyrazolin-5-one into substituted 2*H*-spiro[furo[2,3-*d*]pyrimidine-6,4′-pyrazole]-2,3′,4(2′*H*,3*H*)-triones **2a**–**h** in alcohols under the action of a base–oxidant system.

**Scheme 1:**
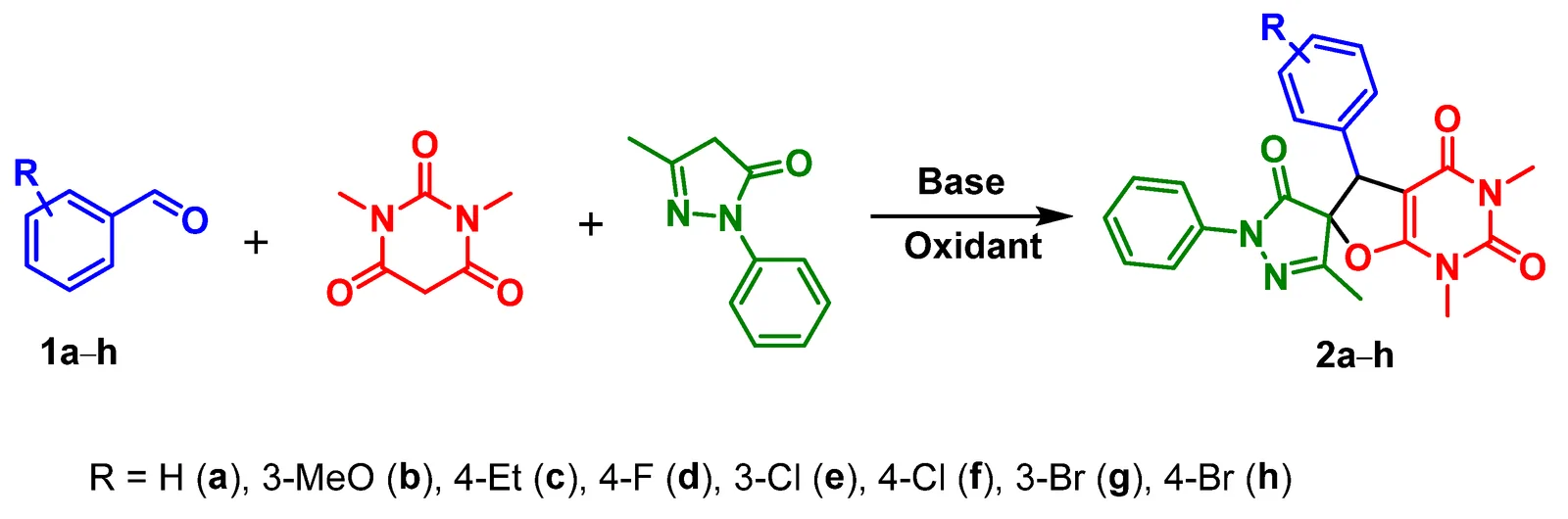
One-pot assembling and cyclization of aldehydes **1a**–**h**, *N*,*N*′-dimethylbarbituric acid, and 3-methyl-1-phenyl-2-pyrazolin-5-one.

At the beginning of our research, we aimed to find the best multicomponent reaction conditions for the Knoevenagel–Michael process with the following oxidative cyclization in the one-pot manner. Thus, we first carried out the direct assembly of benzaldehyde **1a**, *N*,*N*′-dimethylbarbituric acid, and 3-methyl-1-phenyl-2-pyrazolin-5-one into 2*H*-spiro[furo[2,3-*d*]pyrimidine-6,4′-pyrazole]-2,3′,4(2′*H*,3*H*)-trione **2a** using a base–oxidant system (Scheme 1, Table 1).

Previously, we have used a base–molecular halogen system for the one-pot cascade transformation of alkylidenemalononitriles and malononitrile into substituted 1,1,2,2-tetracyanocyclopropanes [27], and also directly from carbonyl compounds and malononitrile [28].

Sodium acetate (NaOAc) is a cost-effective, non-toxic, and widely used weak base catalyst in organic synthesis. It has often been used as a catalyst for aldol condensation of aromatic aldehydes and acid anhydrides (Perkin reaction) [29], and for condensation of hippuric acid with aromatic aldehydes (Erlenmeyer–Plöchl reaction) [30]. We have previously found NaOAc to catalyze multicomponent cyclization of benzaldehydes, malononitrile, and acetone into cis-2,6-diaryl-4-(dicyanomethylidenecyclohexane)-1,1-dicarbonitriles [31]. In this research, NaOAc was found to be a sufficiently good base in the base–*N*-bromosuccinimide (NBS) oxidant system. (Table 1, entry 10).

The only limitation of NBS as a green brominating agent is the formation of succinimide as organic waste and its subsequent removal from the reaction mixture. As an oxidant, NBS has been successfully employed in various reactions for new C–C [32], C–O [33], C–N [34], and C–S [35] bond formation.

In the present research it was found that NBS was the best oxidant for directly assembling benzaldehyde **1a**, *N*,*N*′-dimethylbarbituric acid and 3-methyl-1-phenyl-2-pyrazolin-5-one into spiro[furo[2,3-*d*]pyrimidine-6,4′-pyrazole] **2a** (Table 1, entries 7, 10 and 12–15). *N*-Iodosuccinimide (NIS) and *N*-chlorosuccinimide (NCS) were less efficient in the cyclization process compared to NBS (Table 1, entries 8 and 9). Under the conditions of method **A** with the simultaneous addition of NaOAc and NBS the best result was achieved in EtOH and **2a** was obtained in 68% yield (Table 1, entry 10). Further improvement has been achieved by NaOAc in the reaction mixture. With method **B** spiro[furo[2,3-*d*]pyrimidine-6,4′-pyrazole] **2a** was received in the best 90% yield (Table 1, entry 14).

Under the optimal conditions thus found (ethanol as a solvent, NaOAc–NBS base–oxidant system, method **B** 60 + 60 min reaction time at ambient temperature), compounds **2a**–**h** were formed in 73–90% yields (Table 2).

In all these multicomponent processes, after the end of the reaction, the reaction mixture was filtered out, rinsed with an ice-cold ethanol/water solution (1:1, 2 mL), and dried under reduced pressure.

The structure of all new compounds **2a**–**h** was confirmed by ^1^H, ^13^C NMR, and IR spectroscopy, as well as exact mass spectrometry data. For all compounds, only one set of signals was observed in ^1^H and ^13^C NMR spectra.

**Figure 1:**
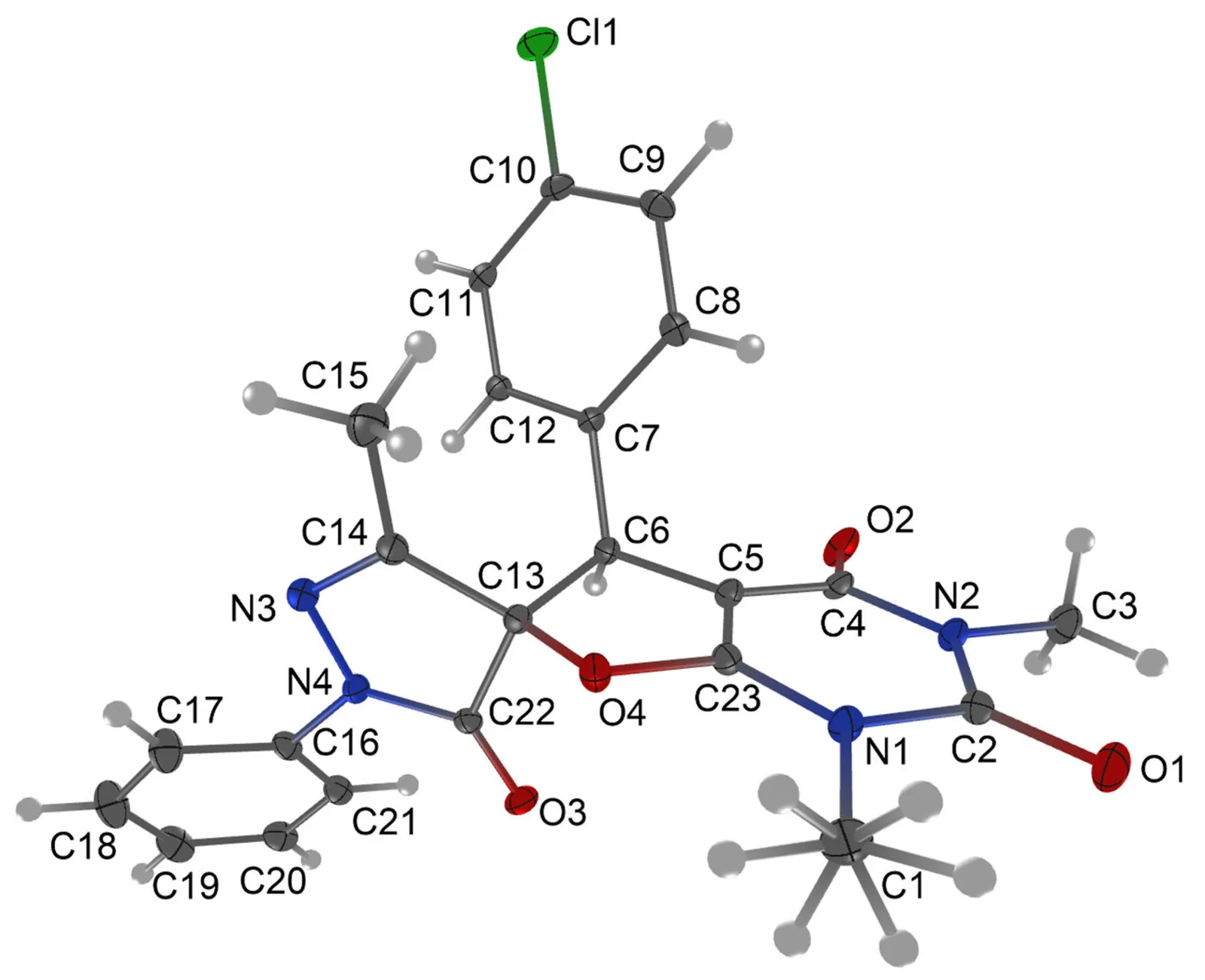
Molecular structure **2f** in the representation of atoms in ellipsoids of anisotropic atomic displacements with a probability of 50%.

The structure of compound **2f** was additionally confirmed by X-ray diffraction data (Figure 1).

**Scheme 2:**
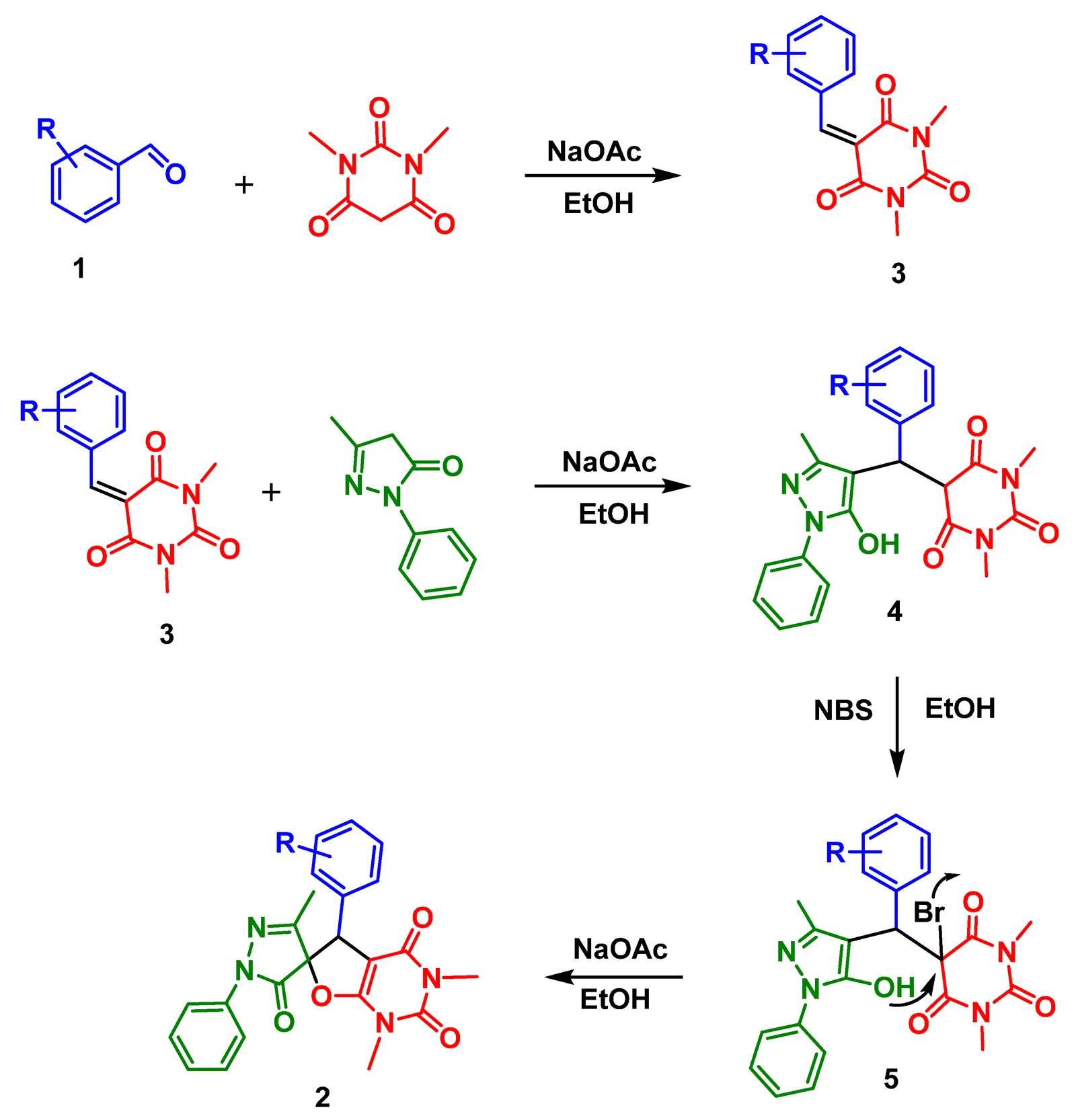
One-pot assembling and cyclization of aldehydes **1**, *N*,*N*′-dimethylbarbituric acid, and 3-methyl-1-phenyl-2-pyrazolin-5-one.

Taking into consideration the above results (Table 1 and Table 2) and the data for the mechanisms of the transformation of carbonyl compounds and CH-acids into spirocyclic heterocyclic compounds, the following mechanism for the transformation of aldehydes **1a**–**h**, *N*,*N*′-dimethylbarbituric acid, and 3-methyl-1-phenyl-2-pyrazolin-5-one into spiro[furo[2,3-*d*]pyrimidine-6,4′-pyrazoles] **2a**–**h** was proposed (Scheme 2).

In the first stage, the NaOAc-catalyzed reaction of aldehyde **1** and *N*,*N*′-dimethylbarbituric acid results in the formation of Koevenagel adduct 5-arylidene-1,3-dimethylpyrimidine-2,4,6(1*H*,3*H*,5*H*)-trione **3**. Further Michael addition of the molecule of 3-methyl-1-phenyl-2-pyrazolin-5-one to the activated double bond of Koevenagel adduct **3** leads to Michael adduct 5-[(5-hydroxy-3-methyl-1-phenyl-1*H*-pyrazol-4-yl)(aryl)methyl]-1,3-dimethylpyrimidine-2,4,6(1*H*,3*H*,5*H*)-trione **4**. Bromination of Michael adduct **4** by NBS affords 5-bromo-5-[(5-hydroxy-3-methyl-1-phenyl-1*H*-pyrazol-4-yl)(phenyl)methyl]-1,3-dimethylpyrimidine-2,4,6(1*H*,3*H*,5*H*)-trione **5** formation. And the last step of the one-pot process is cyclization of bromo-substituted Michael adduct **5** into 2*H*-spiro[furo[2,3-*d*]pyrimidine-6,4′-pyrazole]-2,3′,4(2′*H*,3*H*)-trione **2** in the presence of a base (NaOAc).

## 3. Materials and Methods

All commercially available chemicals and solvents were purchased from Sigma-Aldrich (Burlington, Massachusetts, USA) and Alfa Aesar (Ward Hill, Massachusetts, USA) and used as received without further purification.

Melting points were determined using a Gallenkamp melting-point apparatus (Gallenkamp & Co., Ltd., London, UK) and are uncorrected. ^1^H and ^13^C NMR spectra were recorded in DMSO-*d*_6_ on a Bruker Avance II 300 spectrometer (Bruker Corporation, Billerica, MA, USA) at ambient temperature. Chemical shifts (δ) are reported in parts per million (ppm) relative to tetramethylsilane (TMS) as an internal standard. Signal assignments were made based on the accumulated experience of previous studies and standard chemical shifts for the respective groups. IR spectra were recorded on a Bruker ALPHA-T FT-IR spectrometer (Bruker Corporation, Billerica, MA, USA) using KBr pellets. High-resolution mass spectra (HRMS) were obtained on a Bruker MicrOTOF-Q II spectrometer (Bruker Corporation, Billerica, MA, USA) using electrospray ionization (ESI) in positive ion mode and processed with Bruker Compass DataAnalysis 4.0 software.

### 3.1. Single Crystal X-Ray Diffraction

X-ray diffraction data for compound **2f** were collected on a Bruker Smart APEX II diffractometer (Bruker Corporation, Billerica, MA, USA) using graphite-monochromated MoKα (λ = 0.71073 Å) radiation at 120 K. Frame integration and absorption corrections were performed using the APEX3 software (version APEX3.0.0) package (Bruker AXS Inc., Madison, WI, USA). The structure was solved using the dual-space algorithm (SHELXT program, version 2014/4 [36]) and refined by the least-squares method (SHELXL program, version 2014/6 [37]) with anisotropic displacement parameters for all non-hydrogen atoms. Hydrogen atoms were placed in calculated positions and refined using a riding model. Crystal data for **2f**: monoclinic, space group P2_1_/c, C_23_H_19_ClN_4_O_4_, M = 450.87, a = 18.2275(18), b = 14.3084(14), c = 8.1517(8) Å, β = 91.640(3)°, V = 2125.1(4) Å^3^, Z = 4, dcalc = 1.409 g/cm^3^, μ = 0.219 mm^−1^, 6426 unique reflections collected, 2θ < 61.082°. Final R = 0.0436 [4409 with I > 2σ(I)], wR2 = 0.1145 [all 6426 I], S = 1.079. Crystallographic data for the structure reported in this paper have been deposited with the Cambridge Crystallographic Data Centre (CCDC 2536541).

### 3.2. General Procedure for the Synthesis of Compounds ***2a***–***h***

A solution of arylaldehyde **1** (1 mmol), *N*,*N*′-dimethylbarbituric acid (1 mmol, 0.156 g), 3-methyl-1-phenyl-2-pyrazolin-5-one (1 mmol, 0.174 g), and sodium acetate (1 mmol, 0.082 g) in ethanol (4 mL) was stirred at room temperature for 1 h. Then, *N*-bromosuccinimide (1.3 mmol, 0.231 g) was added, and the mixture was stirred at room temperature for an additional 1 h. After completion of the reaction, the precipitated product was collected by filtration, washed with an ice-cold ethanol/water solution (1:1, 2 mL), and dried under reduced pressure to afford the pure target compounds **2a**–**h**.

### 3.3. Analytical and Spectral Data of Compounds ***2a***–***h***

***1,3,3′-Trimethyl-1′,5-diphenyl-1,5-dihydro-2H-spiro[furo[2,3-d]pyrimidine-6,4′-pyrazole]-2,4,5′(1′H,3H)-trione (2a)*.** Yield: 90%. M.p.: 205–206 °C (EtOH). ^1^H NMR (300 MHz, DMSO-*d*_6_) δ 7.81 (d, ^3^*J* = 8.0 Hz, 2H, C(2)H and C(6)H Ar pyr), 7.51 (t, ^3^*J* = 8.0 Hz, 2H, C(3)H and C(5)H Ar pyr), 7.41–7.22 (m, 6H, C(4)H Ar pyr and 5H Ar from Ph), 5.14 (s, 1H, CH), 3.36 (s, 3H, N-CH_3_), 3.22 (s, 3H, N-CH_3_), 1.39 (s, 3H, CH_3_ pyr) ppm. ^13^C NMR (75 MHz, DMSO-*d*_6_) δ 168.6 (C=O pyr), 161.8 (C^4^=O), 158.4 (C^2^=O), 156.7 (C^1a^), 150.9 (C-CH_3_), 136.8 (C^1^ Ar), 134.4 (C^1^ Ar pyr), 129.1 (2C, C^3^ and C^5^ Ar pyr), 128.8 (C^4^ Ar), 128.6 (2C, C^3^ and C^5^ Ar), 128.2 (2C, C^2^ and C^6^ Ar), 125.8 (C^4^ Ar pyr), 118.8 (2C, C^2^ and C^6^ Ar pyr), 91.0 (C^4a^), 86.2 (C spiro), 52.2 (CH), 29.7 (N-CH_3_), 27.8 (N-CH_3_), 13.8 (CH_3_ pyr) ppm. IR (KBr) ν 2957 (C-H), 1722 (C=O), 1654 (C=O), 1498 (C=C Ar), 1438 (CH_3_), 1310 (C-N), 1190 (C-O-C), 1085 (C-O) cm^−1^. HRMS (ESI) calculated for C_23_H_2_0N_4_O_4_ + H^+^ [M + H^+^]: 417.1557. Found: 417.1561.

***5-(3-Methoxyphenyl)-1,3,3′-trimethyl-1′-phenyl-1,5-dihydro-2H-spiro[furo[2,3-d]pyrimidine-6,4′-pyrazole]-2,4,5′(1′H,3H)-trione (2b).*** Yield: 82%. M.p.: 170–171 °C (EtOH). ^1^H NMR (300 MHz, DMSO-*d*_6_) δ 7.80 (d, ^3^*J* = 8.0 Hz, 2H, C(2)H and C(6)H Ar pyr), 7.50 (t, ^3^*J* = 8.0 Hz, 2H, C(3)H and C(5)H Ar pyr), 7.35–7.23 (m, 2H, C(4)H Ar pyr, C(5)H Ar), 6.92 (d, ^3^*J* = 7.0 Hz, 1H, C(6)H Ar), 6.89–6.81 (m, 2H, C(2)H Ar and C(4)H Ar), 5.09 (s, 1H, CH), 3.70 (s, 3H, OCH_3_), 3.35 (s, 3H, N-CH_3_), 3.21 (s, 3H, N-CH_3_), 1.45 (s, 3H, CH_3_ pyr) ppm. ^13^C NMR (75 MHz, DMSO-*d*_6_*)* δ 168.6 (C=O pyr), 161.8 (C-OCH_3_), 159.4 (2C, C^4^=O and C^2^=O), 158.4 (C^1a^), 156.7 (C-CH_3_), 136.8 (C^1^ Ar), 136.0 (C^1^ Ar pyr), 129.7 (C^5^ Ar), 129.1 (2C, C^3^ and C^5^ Ar pyr), 125.7 (C4 Ar pyr), 120.4 (C^6^ Ar), 118.7 (2C, C^2^ and C^6^ Ar pyr), 114.3 (C^2^ Ar), 113.4 (C^4^ Ar), 90.9 (C^4a^), 86.3 (C spiro), 55.1 (OCH_3_), 52.2 (CH), 29.6 (N-CH_3_), 27.8 (N-CH_3_), 13.9 (CH_3_ pyr) ppm. IR (KBr) ν 2953 (C-H), 1717 (C=O), 1673 (C=O), 1650 (C=O), 1499 (C=C Ar), 1464 (CH_3_), 1308 (C-N), 1191 (C-O-C), 1080 (C-O) cm^−1^. HRMS (ESI) calculated for C_24_H_22_N_4_O_5_ + H^+^ [M + H^+^]: 447.1663. Found: 447.1666.

***5-(4-Ethylphenyl)-1,3,3′-trimethyl-1′-phenyl-1,5-dihydro-2H-spiro[furo[2,3-d]pyrimidine-6,4′-pyrazole]-2,4,5′(1′H,3H)-trione (2c)*.** Yield: 85%. M.p.: 146–147 °C (EtOH). ^1^H NMR (300 MHz, DMSO-*d*_6_) δ 7.80 (d, ^3^*J* = 8.0 Hz, 2H, C(2)H and C(6)H Ar pyr), 7.50 (t, ^3^*J* = 8.1 Hz, 2H, C(3)H and C(5)H Ar pyr), 7.30 (t, ^3^*J* = 7.4 Hz, 1H, C(4)H Ar pyr), 7.20 (d, ^3^*J* = 8.4 Hz, 2H, C(3)H and C(5)H Ar), 7.17 (d, ^3^*J* = 8.4 Hz, 2H, C(2)H and C(6)H Ar), 5.09 (s, 1H, CH), 3.35 (s, 3H, N-CH_3_), 3.20 (s, 3H, N-CH_3_), 2.60 (q, ^3^*J* = 7.5 Hz, 2H, CH_2_ from Et), 1.41 (s, 3H, CH_3_ pyr), 1.17 (t, ^3^*J* = 7.5 Hz, 3H, CH_3_ from Et) ppm. ^13^C NMR (75 MHz, DMSO-*d*_6_) δ 168.6 (C=O pyr), 161.7 (C^4^=O), 158.4 (C^2^=O), 156.8 (C^1a^), 150.9 (C-CH_3_), 144.0 (C-Et), 136.8 (C^1^ Ar), 131.6 (C^1^ Ar pyr), 129.0 (2^C^, C^3^ and C^5^ Ar pyr), 128.1 (2C, C^3^ and C^5^ Ar), 127.9 (2C, C^2^ and C^6^ Ar), 125.7 (C^4^ Ar pyr), 118.7 (2C, C^2^ and C^6^ Ar pyr), 90.9 (C^4a^), 86.3 (C spiro), 52.0 (CH), 29.6 (N-CH_3_), 29.4 (CH_2_ from Et), 27.7 (N-CH_3_), 15.5 (CH_3_ from Et), 13.8 (CH_3_ pyr) ppm. IR (KBr) ν 2966 (C-H), 1756 (C=O), 1722 (C=O), 1686 (C=O), 1499 (C=C Ar), 1438 (CH_3_), 1321 (C-N), 1185 (C-O-C), 1070 (C-O) cm^−1^. HRMS (ESI) calculated for C_25_H_24_N_4_O_4_ + H^+^ [M + H^+^]: 445.1870. Found: 445.1876.

***5-(4-Fluorophenyl)-1,3,3′-trimethyl-1′-phenyl-1,5-dihydro-2H-spiro[furo[2,3-d]pyrimidine-6,4′-pyrazole]-2,4,5′(1′H,3H)-trione (2d).*** Yield: 78%. M.p.: 226–227 °C (EtOH). ^1^H NMR (300 MHz, DMSO-*d*_6_) δ 7.80 (d, ^3^*J* = 8.1 Hz, 2H, C(2)H and C(6)H Ar pyr), 7.51 (t, ^3^*J* = 8.1 Hz, 2H, C(3)H and C(5)H Ar pyr), 7.41–7.27 (m, 3H, C(2)H Ar and C(6)H Ar, C(1)H pyr),7.26–7.17 (m, C(3)H and C(5)H Ar), 5.16 (s, 1H, CH), 3.35 (s, 3H, N-CH_3_), 3.21 (s, 3H, N-CH_3_), 1.44 (s, 3H, CH_3_ pyr) ppm. ^13^C NMR (75 MHz, DMSO-*d*_6_*)* δ 168.5 (C=O pyr), 162.2 (d, ^1^*J* = 220.0 Hz, C^1^ Ar), 161.8 (C^4^=O), 158.5 (C^2^=O), 156.6 (C^1a^), 151.0 (C-CH_3_), 136.8 (C^1^ Ar pyr), 130.8 (C^4^ Ar), 130.4 (d, 2C, ^3^*J* = 8.8 Hz, C^3^ and C^5^ Ar), 129.1 (2C, C^3^ and C^5^ Ar pyr), 125.8 (C^4^ Ar pyr), 118.8 (2C, C^2^ and C^6^ Ar pyr), 115.4 (d, 2C, ^2^*J* = 22.1 Hz, C^2^ and C^6^), 90.9 (C^4a^), 86.3 (C spiro), 51.3 (CH), 29.7 (N-CH_3_), 27.8 (N-CH_3_), 13.9 (CH_3_ pyr) ppm. IR (KBr) ν 2955 (C-H), 1720 (C=O), 1655 (C=O), 1599 (C=C Ar), 1512 (C=C Ar), 1437 (CH_3_), 1310 (C-N), 1191 (C-O-C), 1066 (C-O) cm^−1^. HRMS (ESI) calculated for C_23_H_19_FN_4_O_4_ + H^+^ [M + H^+^]: 435.1463. Found: 435.1461.

***5-(3-Chlorophenyl)-1,3,3′-trimethyl-1′-phenyl-1,5-dihydro-2H-spiro[furo[2,3-d]pyrimidine-6,4′-pyrazole]-2,4,5′(1′H,3H)-trione (2e).*** Yield: 81%. M.p.: 203–204 °C (EtOH). ^1^H NMR (300 MHz, DMSO-*d*_6_) δ 7.79 (d, ^3^*J* = 7.7 Hz, 2H, C(2)H and C(6)H Ar pyr), 7.55–7.45 (m, 3H, C(4)H, C(5)H and C(6)H Ar), 7.44–7.35 (m, 2H, C(2)H Ar and C(4)H Ar pyr), 7.29 (t, ^3^*J* = 7.7 Hz, 2H, C(3)H and C(5)H Ar pyr), 5.16 (s, 1H, CH), 3.34 (s, 3H, N-CH_3_), 3.20 (s, 3H, N-CH_3_), 1.43 (s, 3H, CH_3_ pyr) ppm. ^13^C NMR (75 MHz, DMSO-*d*_6_) δ 168.4 (C=O pyr), 162.0 (C^4^=O), 158.4 (C^2^=O), 156.2 (C^1a^), 150.9 (C-CH_3_), 137.2 (C^1^ Ar), 136.8 (C^1^ Ar pyr), 133.4 (C-Cl), 130.4 (C5 Ar), 129.0 (2C, C^3^ and C5 Ar pyr), 128.4 (C^2^ Ar), 128.2 (C^6^ Ar), 127.2 (C^4^ Ar), 125.7 (C^4^ Ar pyr), 118.8 (2C, C^2^ and C^6^ Ar pyr), 90.8 (C^4a^), 86.0 (C spiro), 51.4 (CH), 29.7 (N-CH_3_), 27.8 (N-CH_3_), 13.9 (CH_3_ pyr) ppm. IR (KBr) ν 2956 (C-H), 1737 (C=O), 1719 (C=O), 1655 (C=O), 1499 (C=C Ar), 1436 (CH_3_), 1307 (C-N), 1190 (C-O-C), 1084 (C-O) cm^−1^. HRMS (ESI) calculated for C_23_H_19_ClN_4_O_4_ + Na^+^ [M + Na^+^]: 475.0958 (^37^Cl), 473.0987 (^35^Cl). Found: 475.0964 (^37^Cl), 473.0993 (^35^Cl).

***5-(4-Chlorophenyl)-1,3,3′-trimethyl-1′-phenyl-1,5-dihydro-2H-spiro[furo[2,3-d]pyrimidine-6,4′-pyrazole]-2,4,5′(1′H,3H)-trione (2f)*.** Yield: 85%. M.p.: 202–203 °C (EtOH). ^1^H NMR (300 MHz, DMSO-*d*_6_) δ 7.79 (d, ^3^*J* = 8.0 Hz, 2H, C(2)H and C(6)H Ar pyr), 7.50 (t, ^3^*J* = 8.0 Hz, 2H, C(3)H and C(5)H Ar pyr), 7.45 (d, ^3^*J* = 8.4 Hz, 2H, C(3)H and C(5)H Ar), 7.39–7.25 (m, 3H, C(4)H Ar pyr, C(2)H and C(6)H Ar), 5.15 (s, 1H, CH), 3.35 (s, 3H, N-CH_3_), 3.20 (s, 3H, N-CH_3_), 1.45 (s, 3H, CH_3_ pyr) ppm. ^13^C NMR (75 MHz, DMSO-d6) δ 168.4 (C=O pyr), 161.9 (C^4^=O), 158.4 (C^2^=O), 156.4 (C^1a^), 150.9 (C-CH^3^), 136.8 (C^1^ Ar), 133.5 (C^1^ Ar pyr), 133.1 (C-Cl), 130.2 (2C, C^3^ and C^5^ Ar), 129.1 (2C, C^3^ and C^5^ Ar pyr), 128.6 (2C, C^2^ and C^6^ Ar), 125.8 (C^4^ Ar pyr), 118.8 (2C, C^2^ and C^6^ Ar pyr), 90.8 (C^4a^), 86.1 (C spiro), 51.4 (CH), 29.7 (N-CH_3_), 27.8 (N-CH_3_), 14.0 (CH_3_ pyr) ppm. IR (KBr) ν 2957 (C-H), 1724 (C=O), 1660 (C=O), 1497 (C=C Ar), 1437 (CH_3_), 1309 (C-N), 1193 (C-O-C), 1090 (C-O) cm^−1^. HRMS (ESI) calculated for C_23_H_19_ClN_4_O_4_ + H^+^ [M + H^+^]: 453.1138 (^37^Cl), 451.1168 (^35^Cl). Found: 453.1146 (^37^Cl), 451.1175 (^35^Cl).

***5-(3-Bromophenyl)-1,3,3′-trimethyl-1′-phenyl-1,5-dihydro-2H-spiro[furo[2,3-d]pyrimidine-6,4′-pyrazole]-2,4,5′(1′H,3H)-trione (2g)*.** Yield: 73%. M.p.: 202–203 °C (EtOH). ^1^H NMR (300 MHz, DMSO-*d*_6_) δ 7.80 (d, ^3^*J* = 8.1 Hz, 2H, C(2)H and C(6)H Ar pyr), 7.65–7.45 (m, 4H, C(4)H and C(5)H Ar, C(3)H and C(5)H Ar pyr), 7.40–7.25 (m, 3H, C(4)H Ar pyr, C(2)H and C(6)H Ar), 5.16 (s, 1H, CH), 3.34 (s, 3H, N-CH_3_), 3.21 (s, 3H, N-CH_3_), 1.44 (s, 3H, CH_3_ pyr) ppm. ^13^C NMR (75 MHz, DMSO-d6) δ 168.4 (C=O pyr), 162.0 (C4=O), 158.4 (C1a), 156.2 (C2=O), 150.9 (C-CH_3_), 137.5 (C^1^ Ar), 136.8 (C^1^ Ar pyr), 131.3 (C^5^ Ar), 131.1 (C^4^ Ar), 130.7 (C^2^ Ar), 129.1 (2C, C^3^ and C^5^ Ar pyr), 127.5 (C^6^ Ar), 125.8 (C^4^ Ar pyr), 122.0 (C-Br), 118.8 (2C, C^2^ and C^6^ Ar pyr), 90.8 (C^4a^), 86.0 (C spiro), 51.4 (CH), 29.7 (N-CH_3_), 27.8 (N-CH_3_), 13.9 (CH_3_ pyr) ppm. IR (KBr) ν 2955 (C-H), 1722 (C=O), 1655 (C=O), 1498 (C=C Ar), 1436 (CH_3_), 1306 (C-N), 1191 (C-O-C), 1080 (C-O) cm^−1^. HRMS (ESI) calculated for C_23_H_19_BrN_4_O_4_ + H^+^ [M + H^+^]: 497.0642 (^81^Br), 495.0662 (^79^Br). Found: 497.0636 (^81^Br), 495.0654 (^79^Br).

***5-(4-Bromophenyl)-1,3,3′-trimethyl-1′-phenyl-1,5-dihydro-2H-spiro[furo[2,3-d]pyrimidine-6,4′-pyrazole]-2,4,5′(1′H,3H)-trione (2h)*.** Yield: 78%. M.p.: 142–143 °C (EtOH). ^1^H NMR (300 MHz, DMSO-*d*_6_) δ 7.79 (d, ^3^*J* = 8.0 Hz, 2H, C(2)H and C(6)H Ar pyr), 7.58 (d, ^3^*J* = 8.4 Hz, 2H, C(3)H and C(5)H Ar), 7.50 (t, ^3^*J* = 8.0 Hz, 2H, C(3)H and C(5)H Ar pyr), 7.35–7.23 (m, 3H, C(4)H Ar pyr, C(2)H and C(6)H Ar), 5.14 (s, 1H, CH), 3.35 (s, 3H, N-CH_3_), 3.20 (s, 3H, N-CH_3_), 1.46 (s, 3H, CH_3_ pyr) ppm. ^13^C NMR (75 MHz, DMSO-*d*_6_) δ 168.4 (C=O pyr), 161.9 (C^4^=O), 159.4 (C^1a^), 158.4 (C2=O), 156.4 (C-CH_3_), 136.8 (C^1^ Ar), 133.9 (C^1^ Ar pyr), 131.5 (2C, C^3^ and C^5^ Ar), 130.6 (2C, C^2^ and C^6^ Ar), 129.1 (2C, C^3^ and C^5^ Ar pyr), 125.8 (C^4^ Ar pyr), 121.6 (C-Br), 118.8 (2C, C^2^ and C^6^ Ar pyr), 90.7 (C^4a^), 86.0 (C spiro), 51.5 (CH), 29.7 (N-CH_3_), 27.8 (N-CH_3_), 14.0 (CH_3_ pyr) ppm. IR (KBr) ν 2956 (C-H), 1719 (C=O), 1683 (C=O), 1497 (C=C Ar), 1441 (CH3), 1314 (C-N), 1187 (C-O-C), 1069 (C-O) cm^−1^. HRMS (ESI) calculated for C_23_H_19_BrN_4_O_4_ + Na^+^ [M + Na^+^]: 519.0461 (^81^Br), 517.0482 (^79^Br). Found: 519.0468 (^81^Br), 517.0489 (^79^Br).

## 4. Conclusions

The new multicomponent Knoevenagel–Michael reaction with the following NBS-induced cyclization was found. We efficiently accomplished at ambient temperature a one-pot process assembly of arylaldehydes, *N*,*N*′-dimethylbarbituric acid, and 3-methyl-1-phenyl-2-pyrazolin-5-one with the selective formation of substituted 2*H*-spiro[furo[2,3-*d*]pyrimidine-6,4′-pyrazole]-2,3′,4(2′*H*,3*H*)-triones containing three heterocyclic rings in 73–90% yields.

This reaction is a simple and facile route to the substituted 2*H*-spiro[furo[2,3-*d*]pyrimidine-6,4′-pyrazole]-2,3′,4(2′*H*,3*H*)-triones, which are promising compounds for different biomedical applications, among them anticonvulsants, anti-AIDS agents, and anti-inflammatory remedies, using reasonable and non-expensive starting materials.

This synthetic multicomponent procedure does not require heating, long reaction times, or complex equipment. It is easy to carry out, and the procedure for isolating the final compounds is very simple. Mild and facile conditions of this chemical multicomponent process and the simple reasonable isolation procedure allow excellent substance yields. All these advantages make this new multicomponent method valuable for the synthesis of new potential drug libraries and biologically active compounds, and in industry.

## Figures and Tables

**Table 1:** One-pot assembling and cyclization of aldehyde **1a,** *N*,*N*′-dimethylbarbituric acid, and 3-methyl-1-phenyl-2-pyrazolin-5-one.

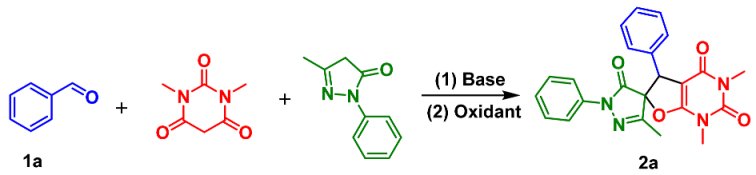						
Entry	Method	Base	Solvent	Oxidant	Time/min	Yield of 2a (%) ^a^
1	**A**	KOH	EtOH	I_2_	60	32
2	**A**	KOH	EtOH	Br_2_	60	36
3	**A**	NaOH	EtOH	I_2_	60	35
4	**A**	NaOH	EtOH	Br_2_	60	42
5	**A**	NaOAc	EtOH	I_2_	60	40
6	**A**	NaOAc	EtOH	Br_2_	60	46
7	**A**	NaOH	EtOH	NBS	60	55
8	**A**	NaOH	EtOH	NIS	60	52
9	**A**	NaOH	EtOH	NCS	60	47
10	**A**	NaOAc	EtOH	NBS	60	68
11	**A**	NaOAc	EtOH	NIS	60	63
12	**A**	NaOAc	MeOH	NBS	60	61
13	**A**	NaOAc	*i*-PrOH	NBS	60	56
14	**B**	NaOAc	EtOH	NBS	60 + 60	90
15	**B**	NaOAc	EtOH	NBS	60 + 90	85

^a^ Benzaldehyde **1a** (1 mmol), *N*,*N*′-dimethylbarbituric acid (1 mmol), 3-methyl-1-phenyl-2-pyrazolin-5-one (1 mmol), base (1 mmol) and oxidant (1.3 mmol) were stirred in solvent (4 mL) at ambient temperature (60 min). The final compound **2a** was isolated by filtration (Method A); benzaldehyde **1a** (1 mmol), *N*,*N*′-dimethylbarbituric acid (1 mmol), 3-methyl-1-phenyl-2-pyrazolin-5-one (1 mmol), and base (1 mmol) were stirred in EtOH (4 mL) at ambient temperature (60 min). Then NBS (1.3 mmol) was added and stirring was continued for another 60 min. The final compound **2a** was isolated by filtration (Method B).

**Table 2:** One-pot assembling and cyclization of aldehyde **1a**–**h,** *N*,*N*′-dimethylbarbituric acid, and 3-methyl-1-phenyl-2-pyrazolin-5-one.

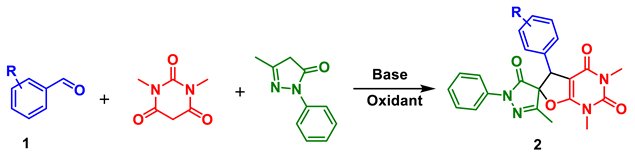
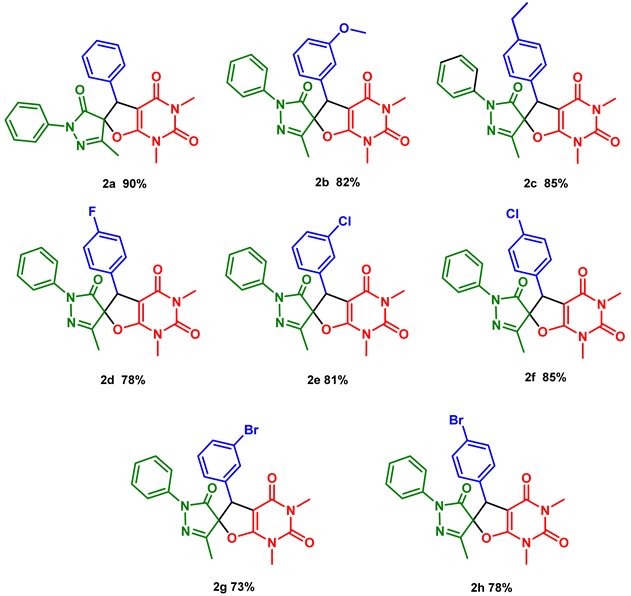

Benzaldehydes **1a**–**h** (1 mmol), *N*,*N*′-dimethylbarbituric acid (1 mmol), 3-methyl-1-phenyl-2-pyrazolin-5-one (1 mmol), and NaOAc (1 mmol) were stirred in EtOH (4 mL) at ambient temperature (60 min). Then NBS (1.3 mmol) was added and stirring was continued for another 60 min. The final compounds **2a**–**h** were isolated by filtration.

## Data Availability

All data that supports the findings of this study is available in the published article and the Supporting Information to this article. Crystallographic data for the structure reported in this paper have been deposited with the Cambridge Crystallographic Data Centre (CCDC 2536541). Copies of the data can be obtained free of charge via https://www.ccdc.cam.ac.uk/structures/ (accessed on 5 July 2026).

## Supplementary Materials

The following supporting information can be downloaded at: https://www.mdpi.com/article/10.3390/molecules31162880/s1, Figure S1: ^1^H NMR spectrum of compound 2**a**, Figure S2: ^13^C NMR spectrum of compound 2**a**, Figure S3: ^1^H NMR spectrum of compound 2**b**, Figure S4: ^13^C NMR spectrum of compound 2**b**, Figure S5: ^1^H NMR spectrum of compound 2**c**, Figure S6: ^13^C NMR spectrum of compound 2**c**, Figure S7: ^1^H NMR spectrum of compound 2**d**, Figure S8: ^13^C NMR spectrum of compound 2**d**, Figure S9: ^1^H NMR spectrum of compound 2**e**, Figure S10: ^13^C NMR spectrum of compound 2**e**, Figure S11: ^1^H NMR spectrum of compound 2**f**, Figure S12: ^13^C NMR spectrum of compound 2**f**, Figure S13: ^1^H NMR spectrum of compound 2**g**, Figure S14: ^13^C NMR spectrum of compound 2**g**, Figure S15: ^1^H NMR spectrum of compound 2**h**, Figure S16: ^13^C NMR spectrum of compound 2**h**, Figure S17: HRMS spectrum of compound 2**a**, Figure S18: HRMS spectrum of compound 2**b**, Figure S19: HRMS spectrum of compound 2**c**, Figure S20: HRMS spectrum of compound 2**d**, Figure S21: HRMS spectrum of compound 2**e**, Figure S22: HRMS spectrum of compound 2**f**, Figure S23: HRMS spectrum of compound 2**g**, Figure S24: HRMS spectrum of compound 2**h**.

## Abbreviations

The following abbreviations are used in this manuscript:

MCR	Multicomponent Reaction
NBS	*N*-Bromosuccinimide
NIS	*N*-Iodosuccinimide
NCS	*N*-Chlorosuccinimide
MMP	Matrix Metalloproteinase
NSAID	Non-Steroidal Anti-Inflammatory Drug
COX	Cyclooxygenase
CNS	Central Nervous System
FDA	Food and Drug Administration (USA)
TPO-RA	Thrombopoietin Receptor Agonist
cITP	Chronic Immune Thrombocytopenia
ALS	Amyotrophic Lateral Sclerosis
NMR	Nuclear Magnetic Resonance
IR	Infrared Spectroscopy
HRMS	Infrared Spectroscopy
ESI	Electrospray Ionization
CCDC	Cambridge Crystallographic Data Centre
TMS	Tetramethylsilane
