# Supplementary material for: Multicomponent Selective and Efficient Synthesis of Spiro[furo[2,3-d]pyrimidine-6,4′-pyrazole] Scaffold Under a Column Chromatography-Free Protocol at Room Temperature
Source: Molecules. 2026 Aug 18;31(16):2880. doi: 10.3390/molecules31162880 (PMC13515434; doi:10.3390/molecules31162880)
Supplement: Supplementary file 1 [file molecules-31-02880-s001.zip › molecules-4449808-supplementary.pdf]

# Supplementary Material:

# Multicomponent Selective and Efficient Synthesis of Spiro[furo[2,3-*d*]pyrimidine-6,4'-pyrazole] Scaffold under a Column Chromatography-Free Protocol at Room Temperature

Michail N. Elinson, Yuliya E. Ryzhkova, Varvara M. Kalashnikova, and Mikhail P. Egorov

*N. D. Zelinsky Institute of Organic Chemistry, Russian Academy of Sciences, 47 Leninsky Pr., Moscow 119991, Russian Federation.* E-mail: elinson@ioc.ac.ru

## Table of Contents

1. The copies of $^1$H NMR and $^{13}$C NMR spectra for compounds **2a-h**.....................................S2
2. The copies of HRMS spectra for compounds **2a-h**...............................................................S10

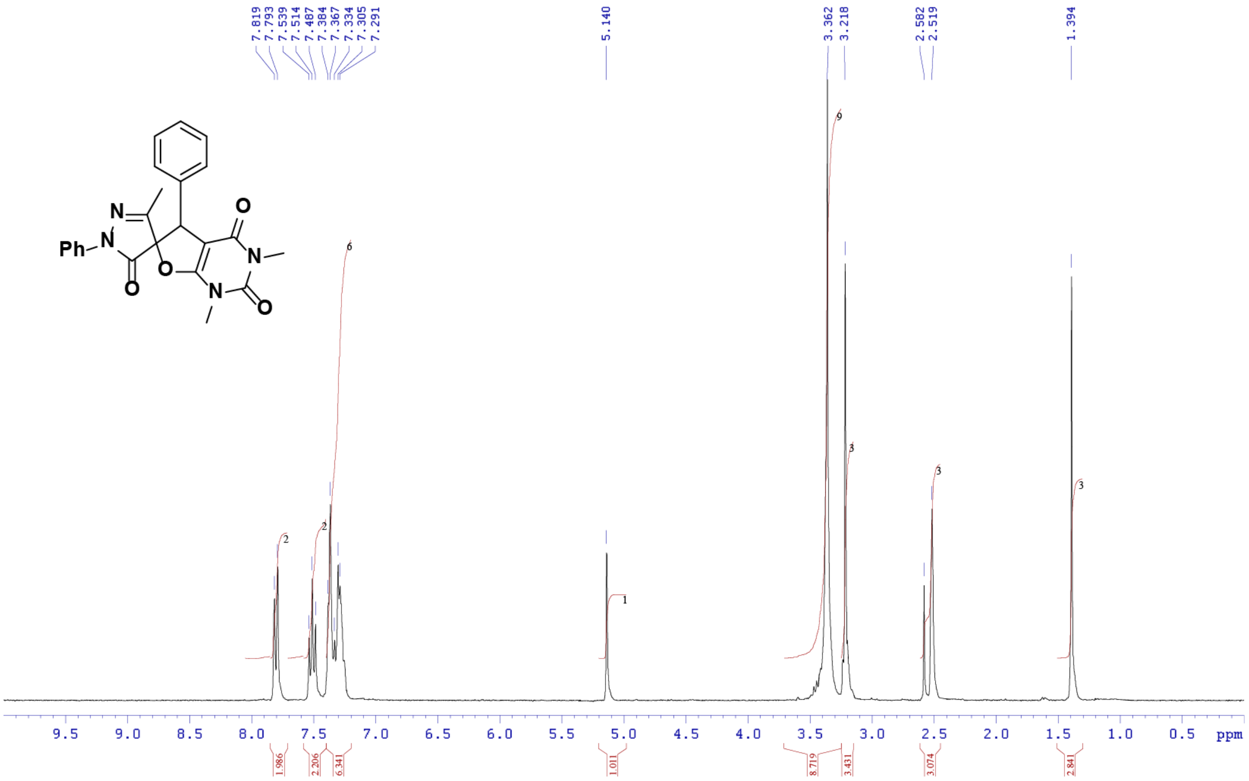


**Figure S1:** $^{1}$H NMR spectrum of compound **2a**

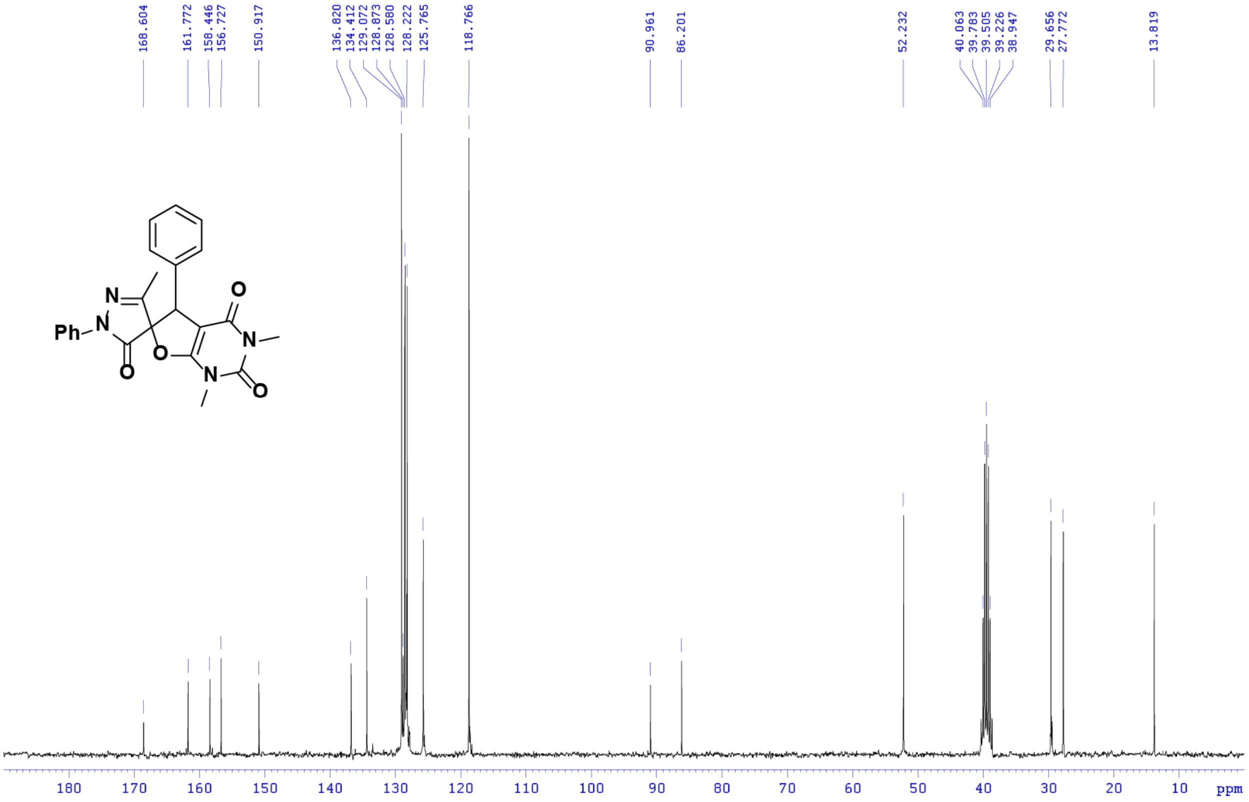


**Figure S2:** $^{13}$C NMR spectrum of compound **2a**

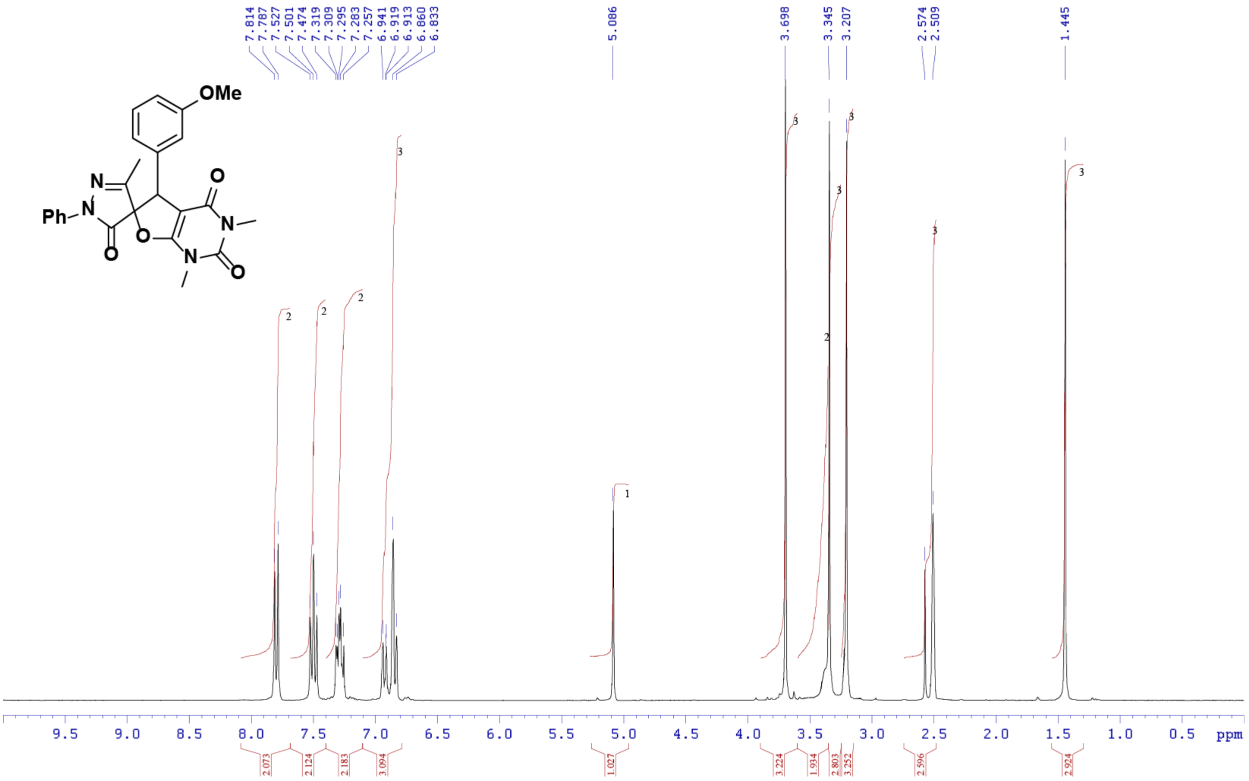


**Figure S3:** $^{1}$H NMR spectrum of compound **2b**

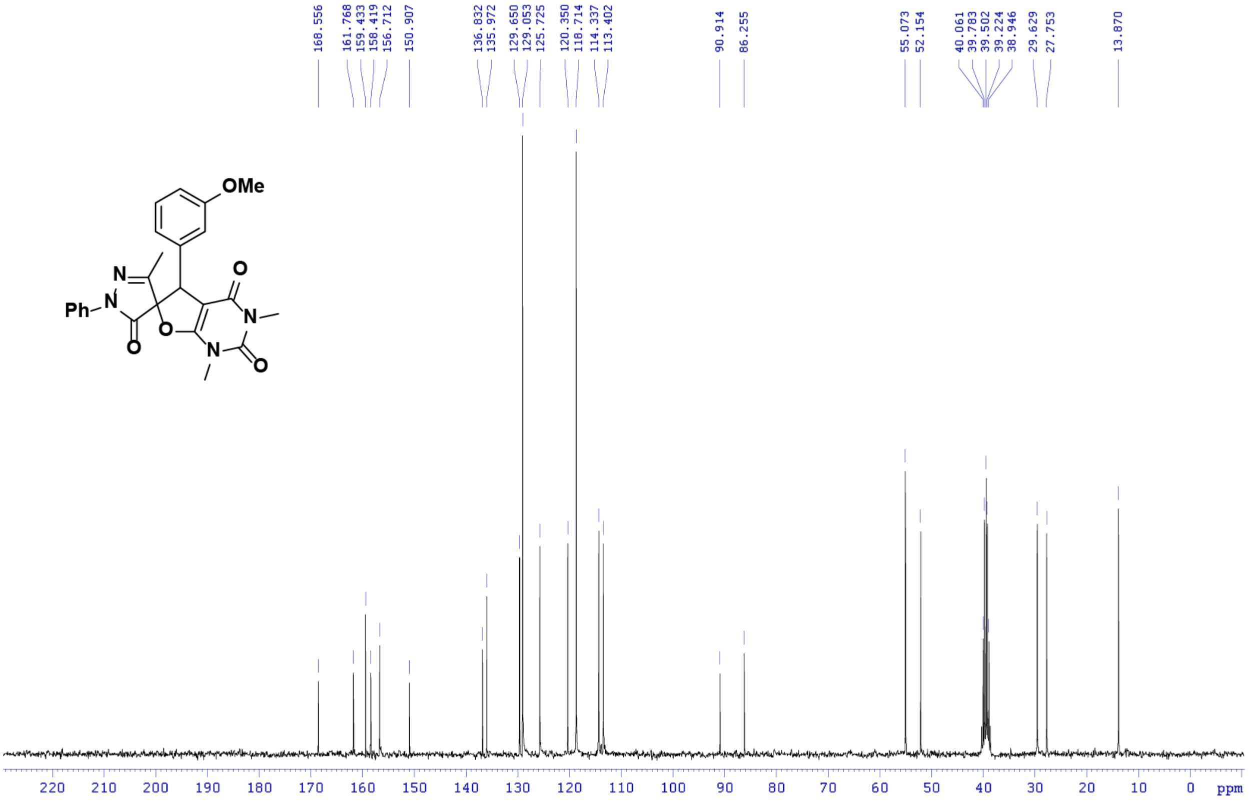


**Figure S4:** $^{13}$C NMR spectrum of compound **2b**

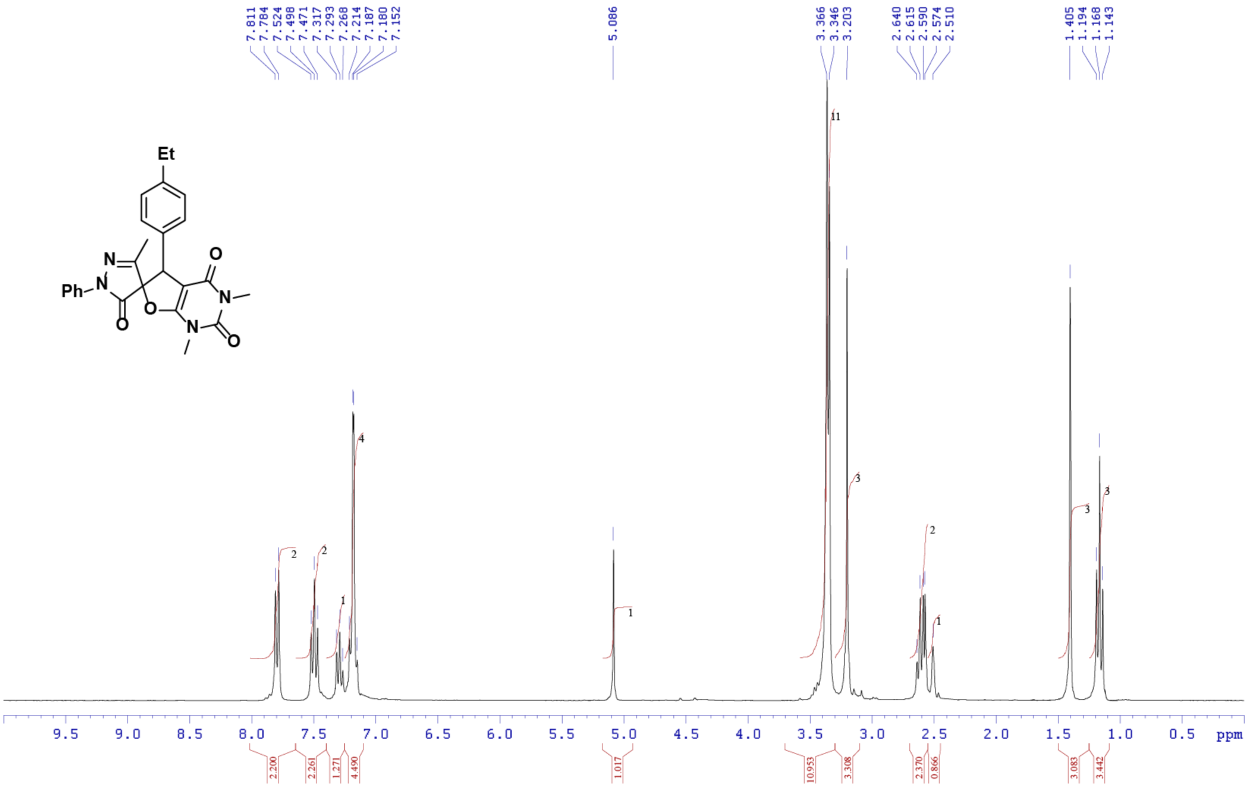

**Figure S5:** $^{1}$H NMR spectrum of compound **2c**

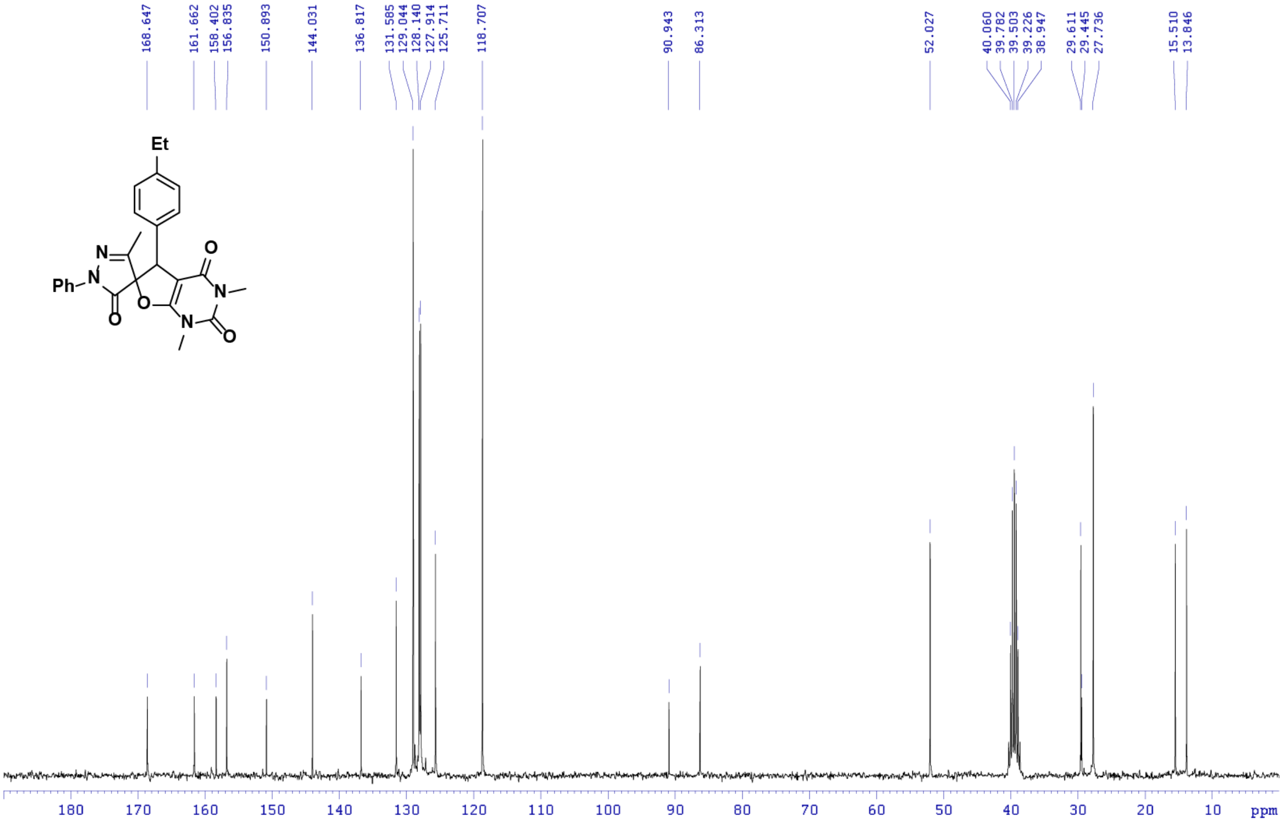

**Figure S6:** $^{13}$C NMR spectrum of compound **2c**

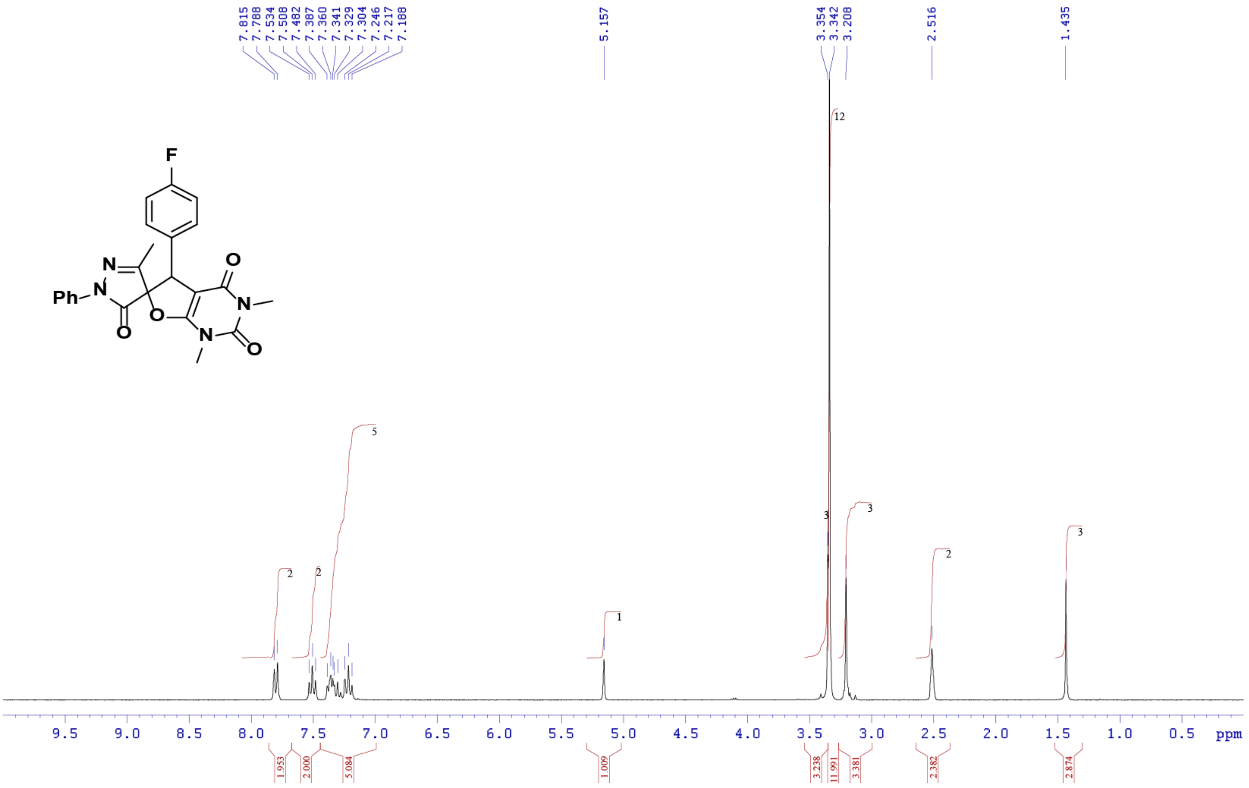


**Figure S7:** $^{1}$H NMR spectrum of compound **2d**

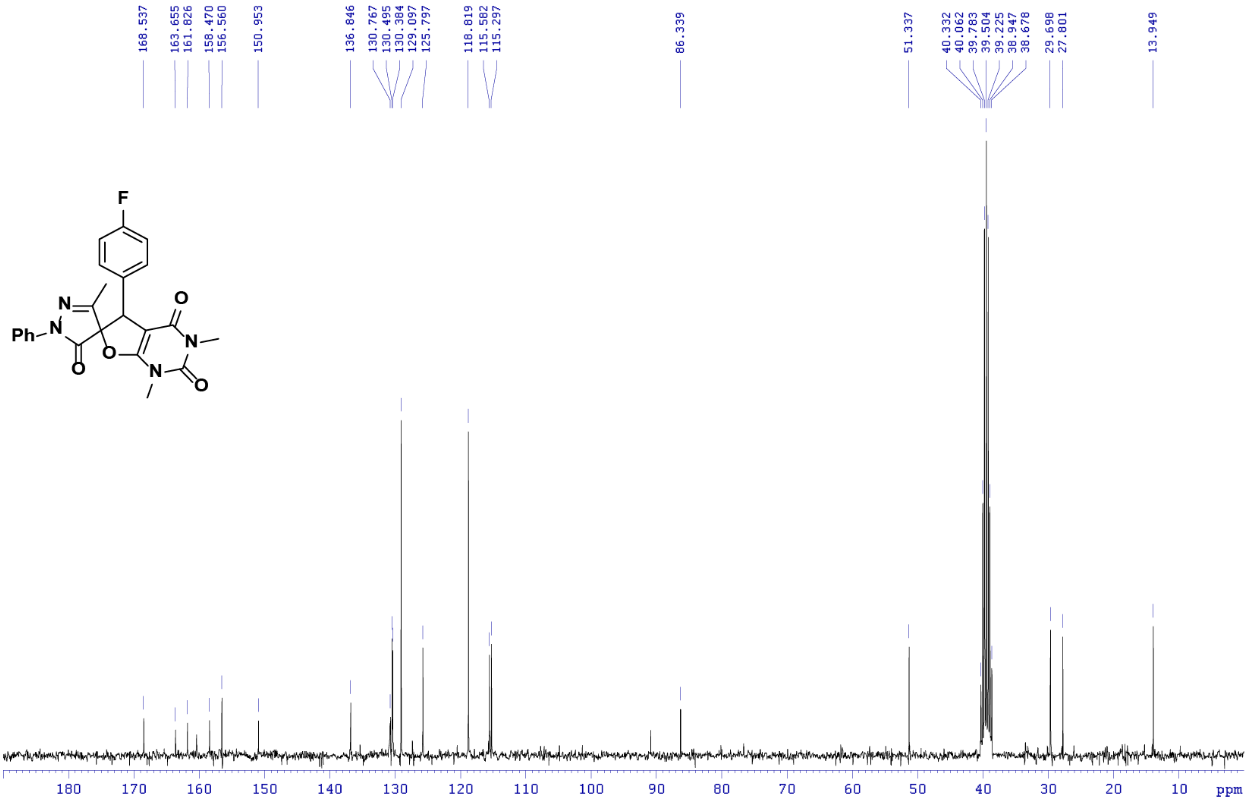


**Figure S8:** $^{13}$C NMR spectrum of compound **2d**

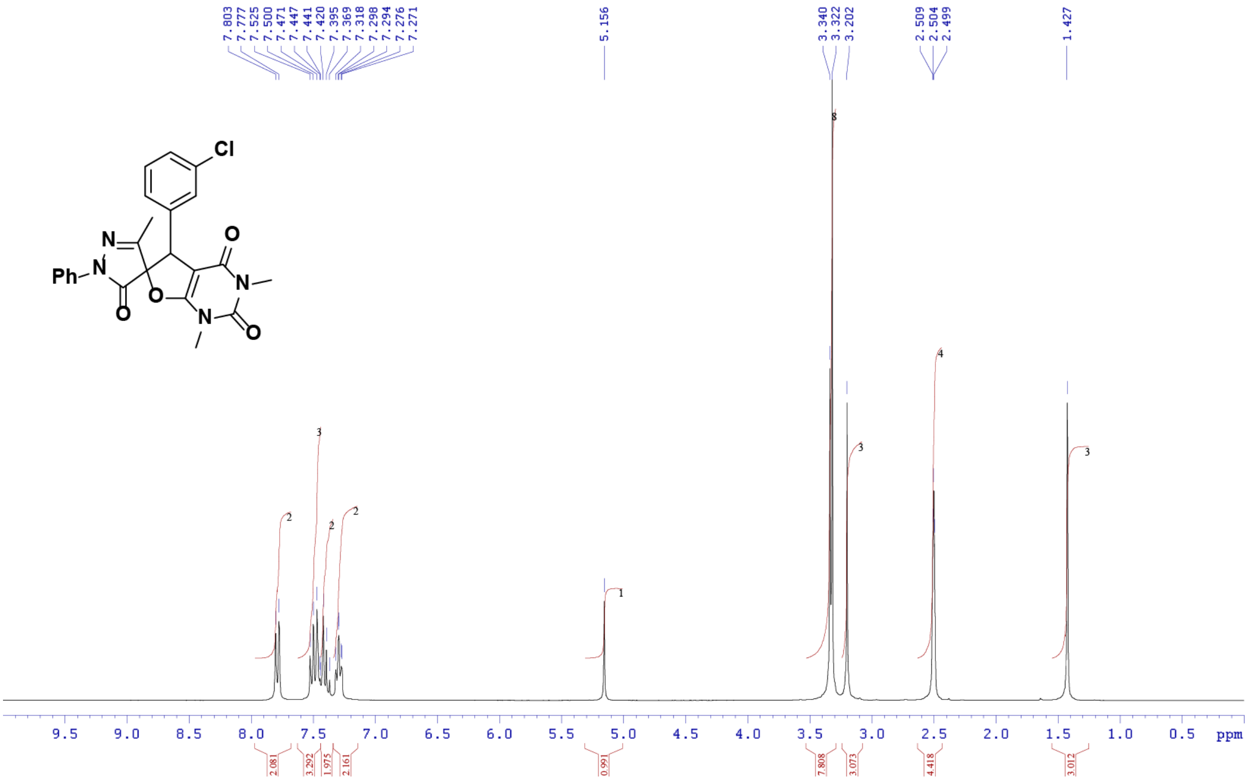


**Figure S9:** $^{1}$H NMR spectrum of compound **2e**

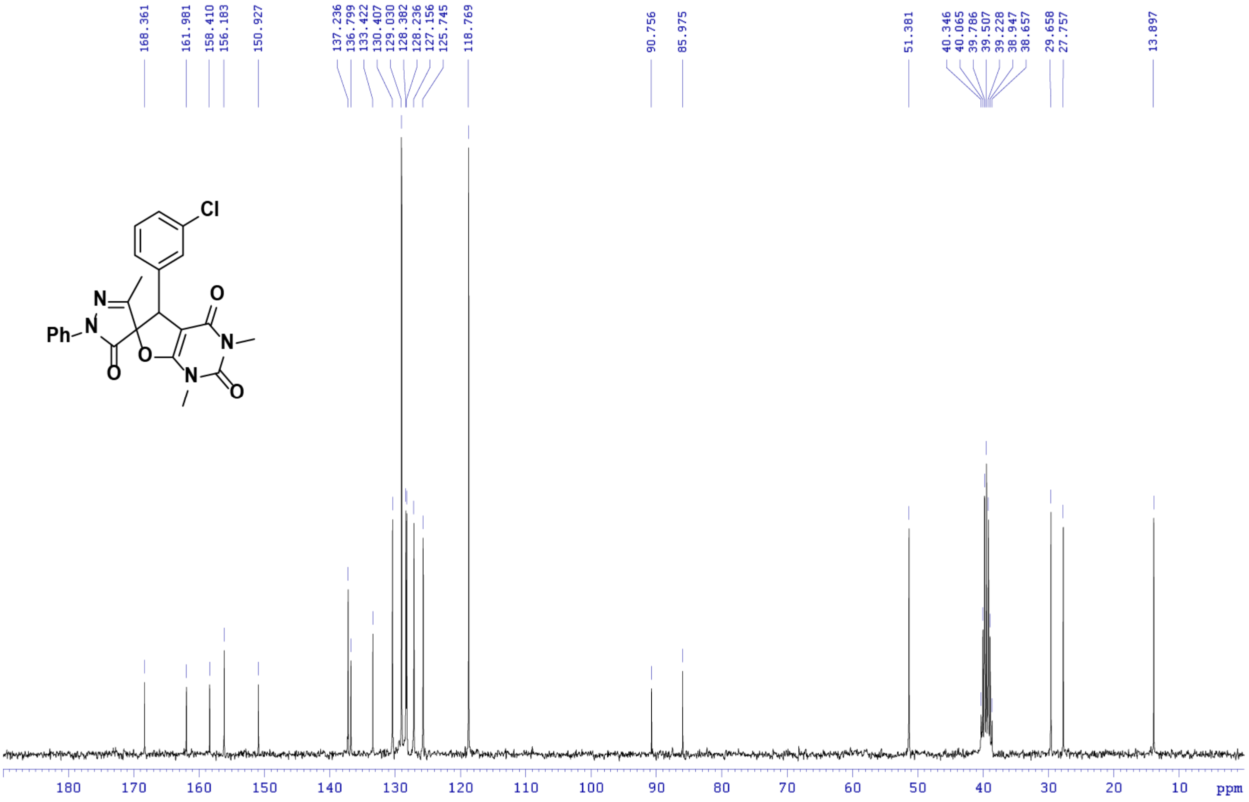


**Figure S10:** $^{13}$C NMR spectrum of compound **2e**

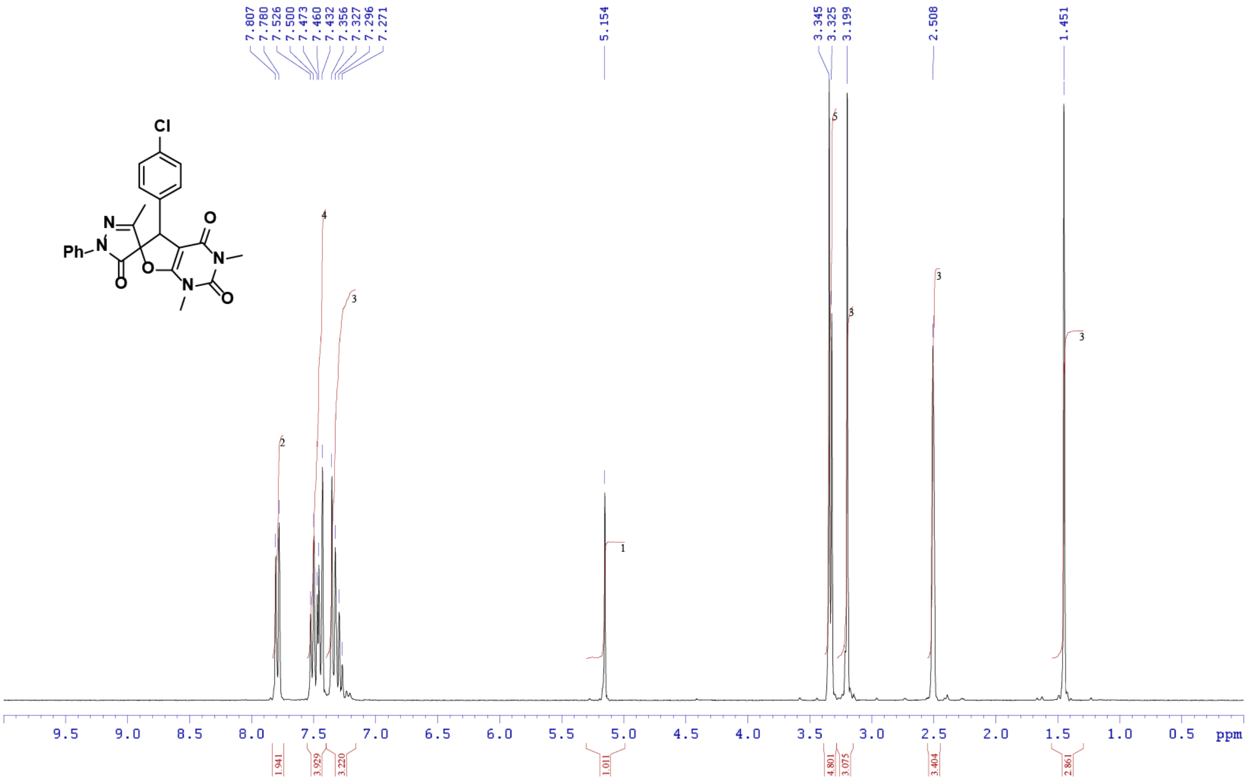


**Figure S11:** $^{1}$H NMR spectrum of compound **2f**

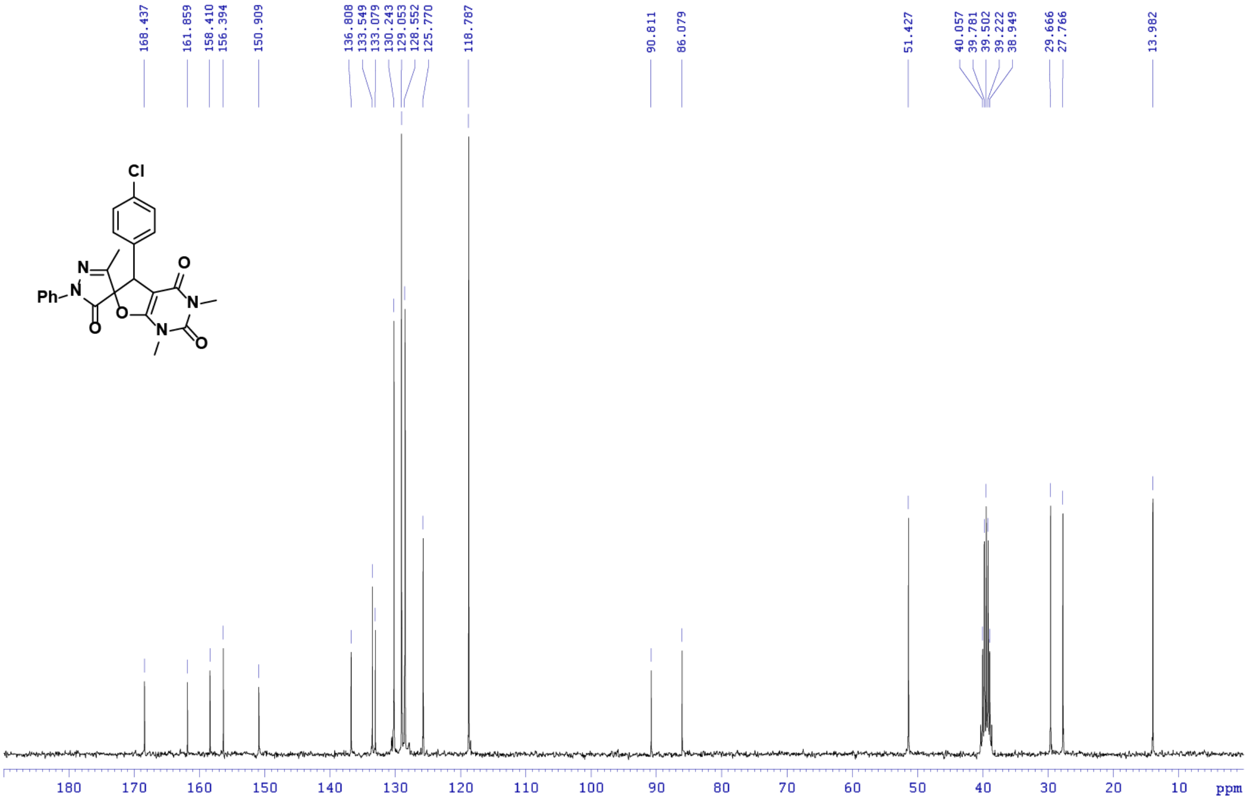


**Figure S12:** $^{13}$C NMR spectrum of compound **2f**

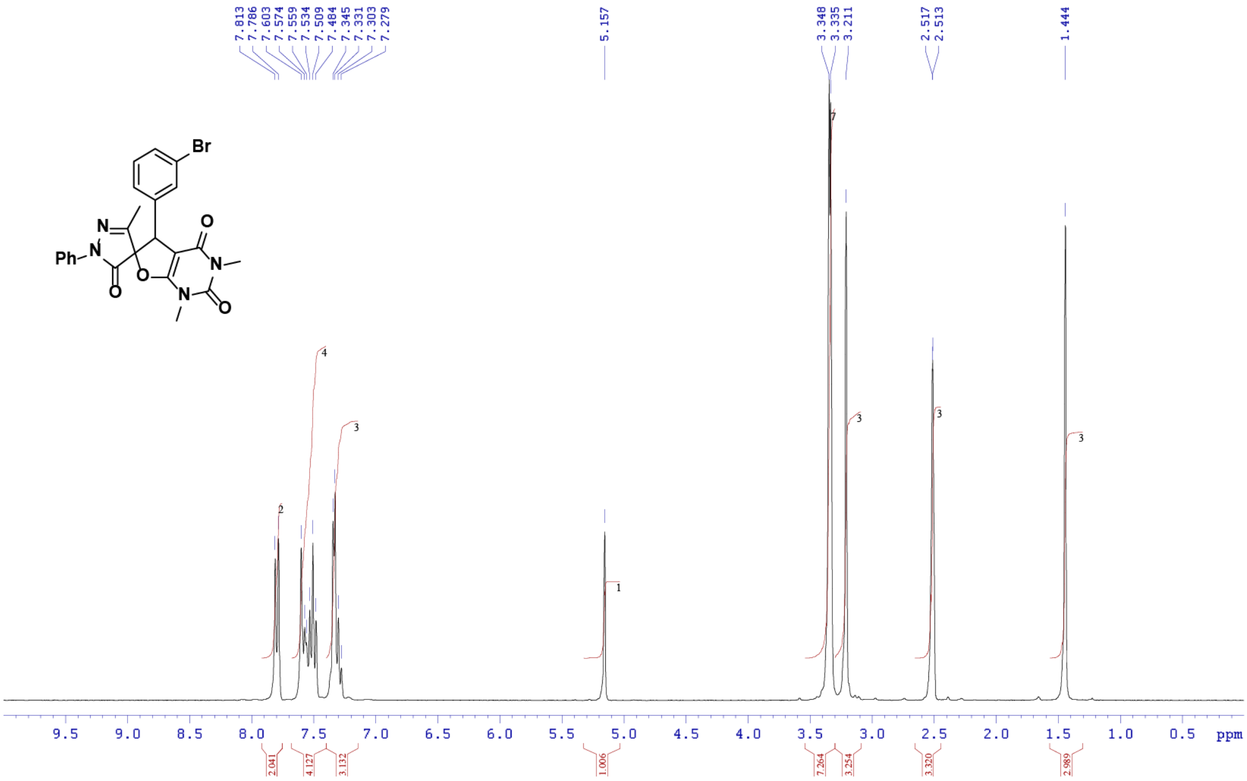


**Figure S13:** $^{1}$H NMR spectrum of compound **2g**

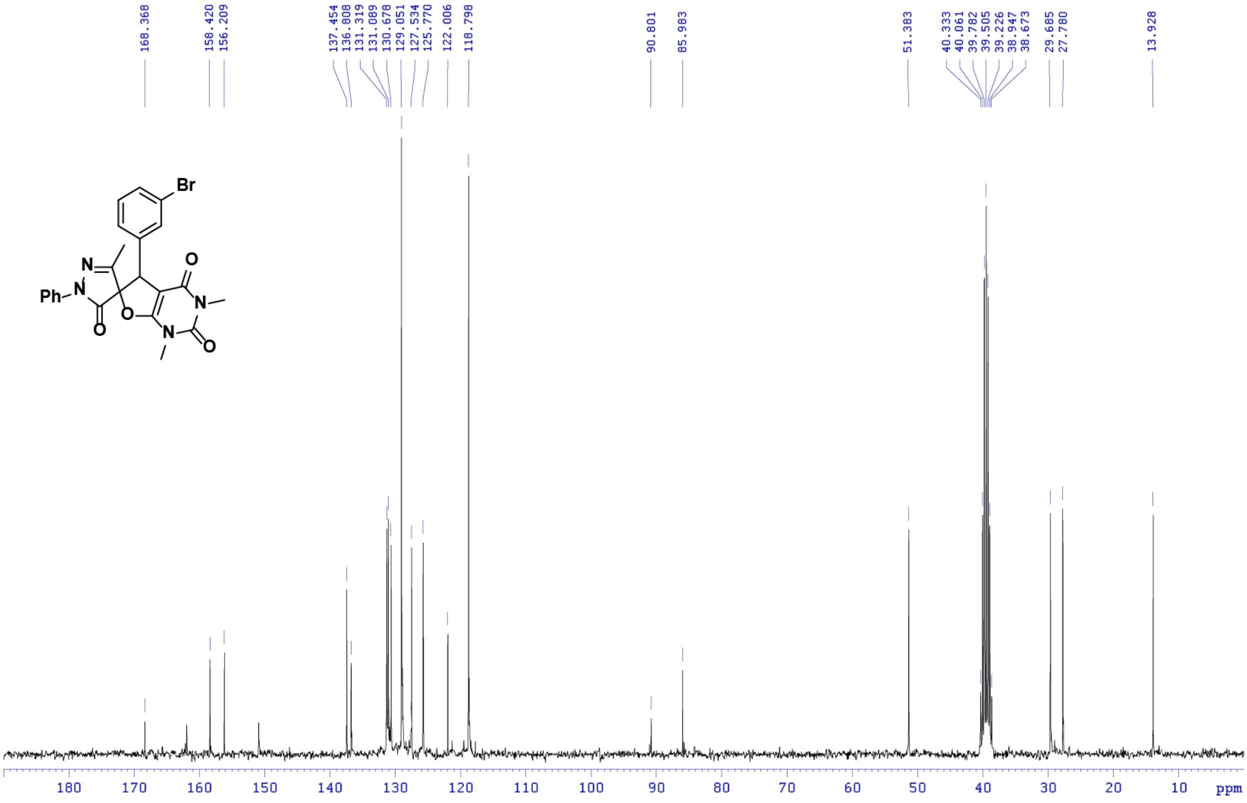


**Figure S14:** $^{13}$C NMR spectrum of compound **2g**

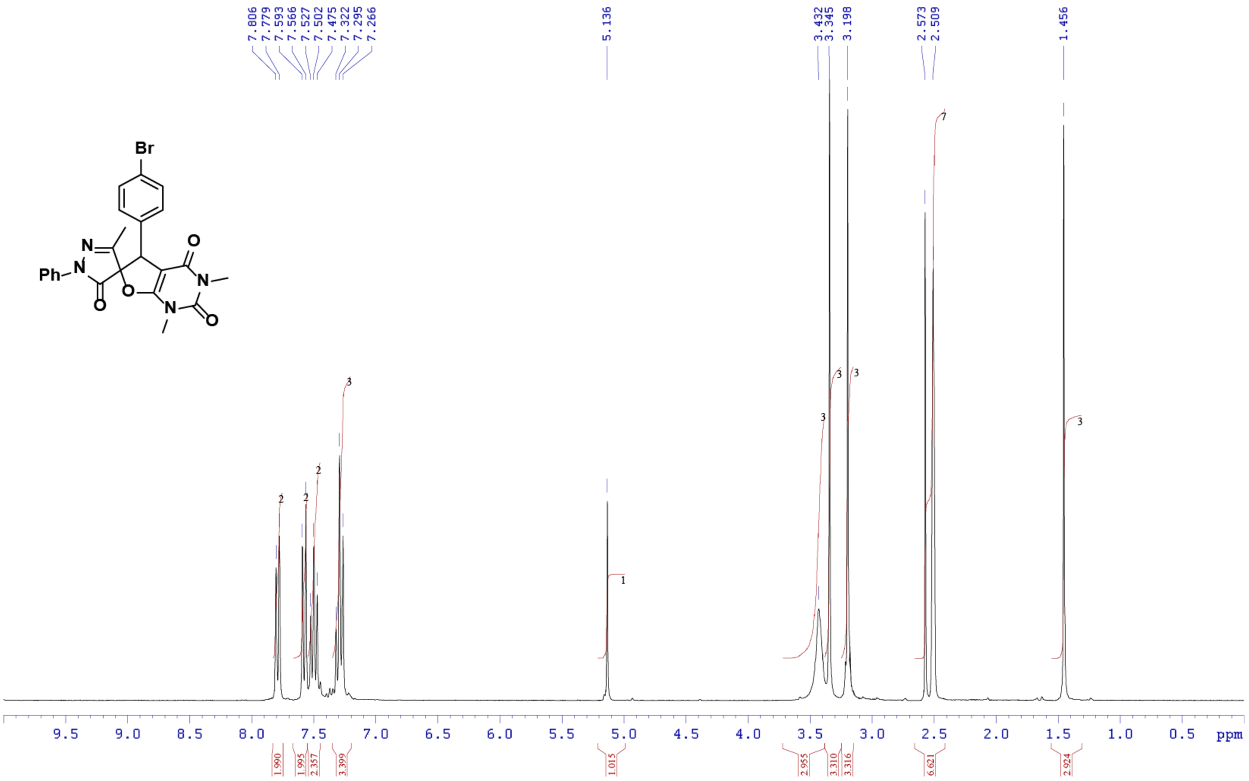


**Figure S15:** $^{1}$H NMR spectrum of compound **2h**

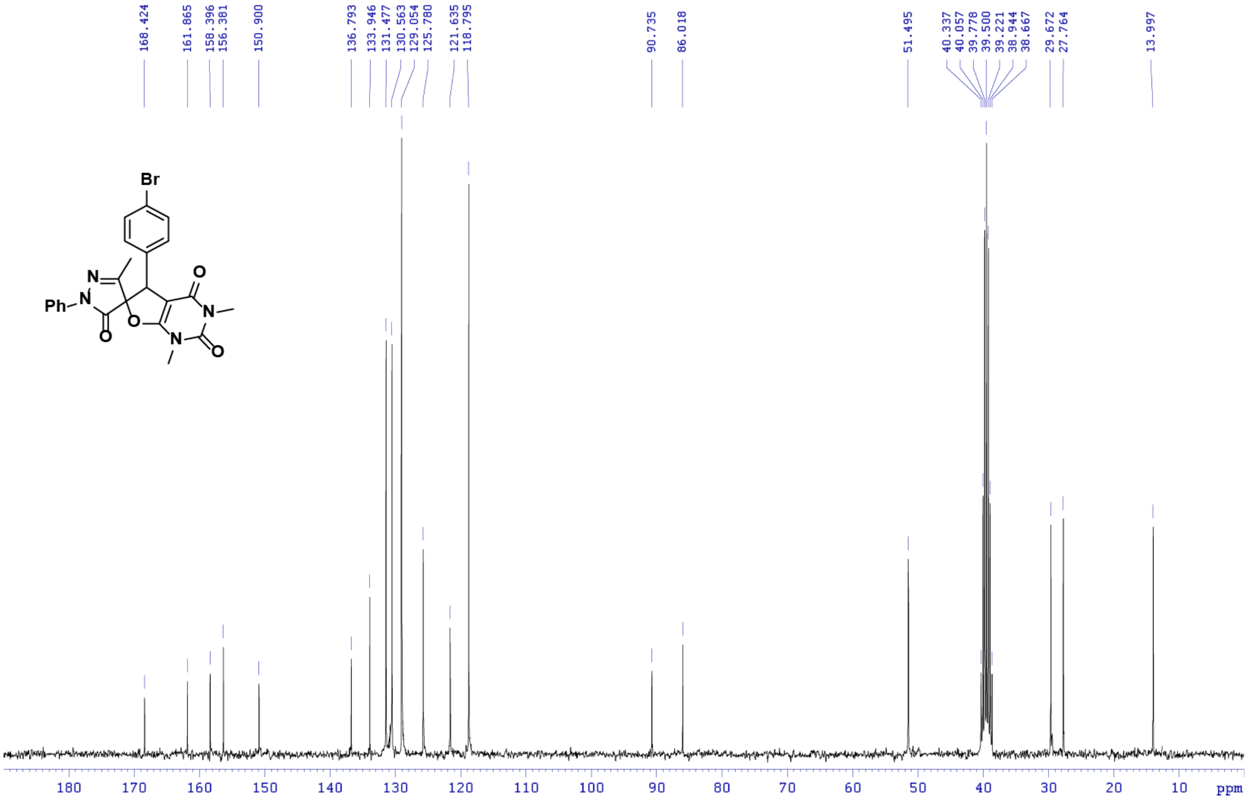


**Figure S16:** $^{13}$C NMR spectrum of compound **2h**

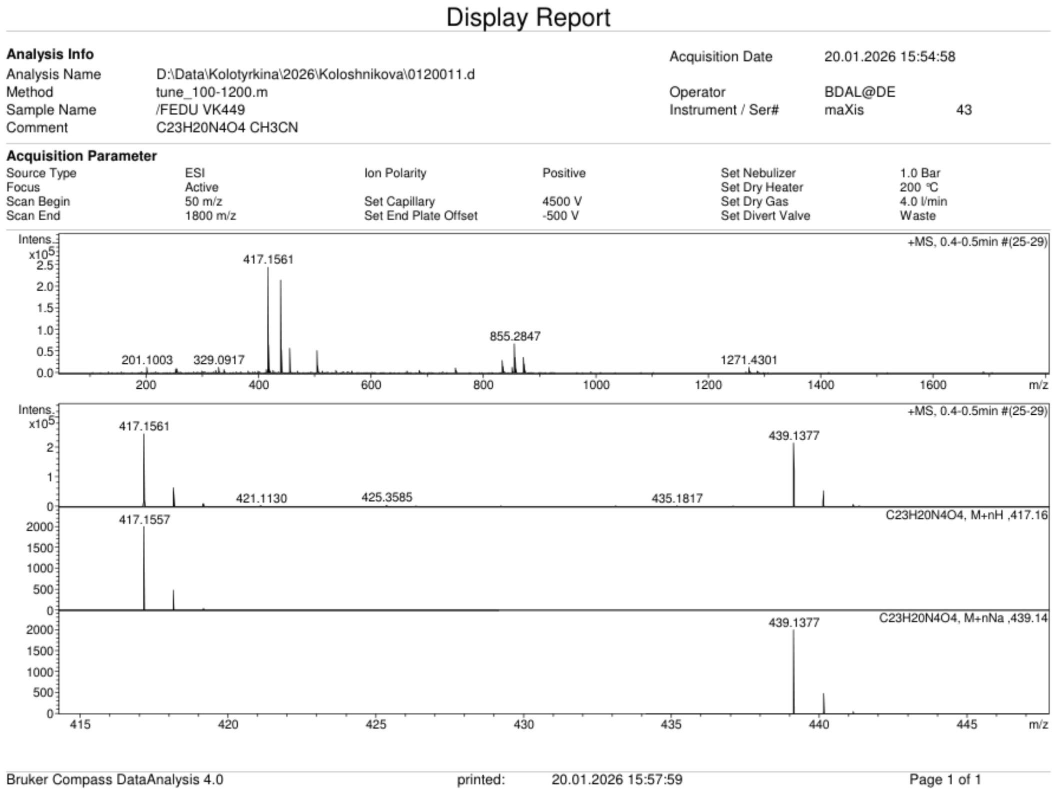


**Figure S17:** HRMS spectrum of compound **2a**

# Display Report

**Analysis Info**

| | | | |
|---|---|---|---|
| Analysis Name | D:\Data\Kolotyrkina\2026\Koloshnikova\0120006.d | Acquisition Date | 20.01.2026 15:34:08 |
| Method | tune_100-1200.m | Operator | BDAL@DE |
| Sample Name | /FEDU VK502 | Instrument / Ser# | maXis 43 |
| Comment | C24H22N4O5 CH3CN | | |

**Acquisition Parameter**

| | | | | | |
|---|---|---|---|---|---|
| Source Type | ESI | Ion Polarity | Positive | Set Nebulizer | 1.0 Bar |
| Focus | Active | | | Set Dry Heater | 200 °C |
| Scan Begin | 50 m/z | Set Capillary | 4500 V | Set Dry Gas | 4.0 l/min |
| Scan End | 1800 m/z | Set End Plate Offset | -500 V | Set Divert Valve | Waste |

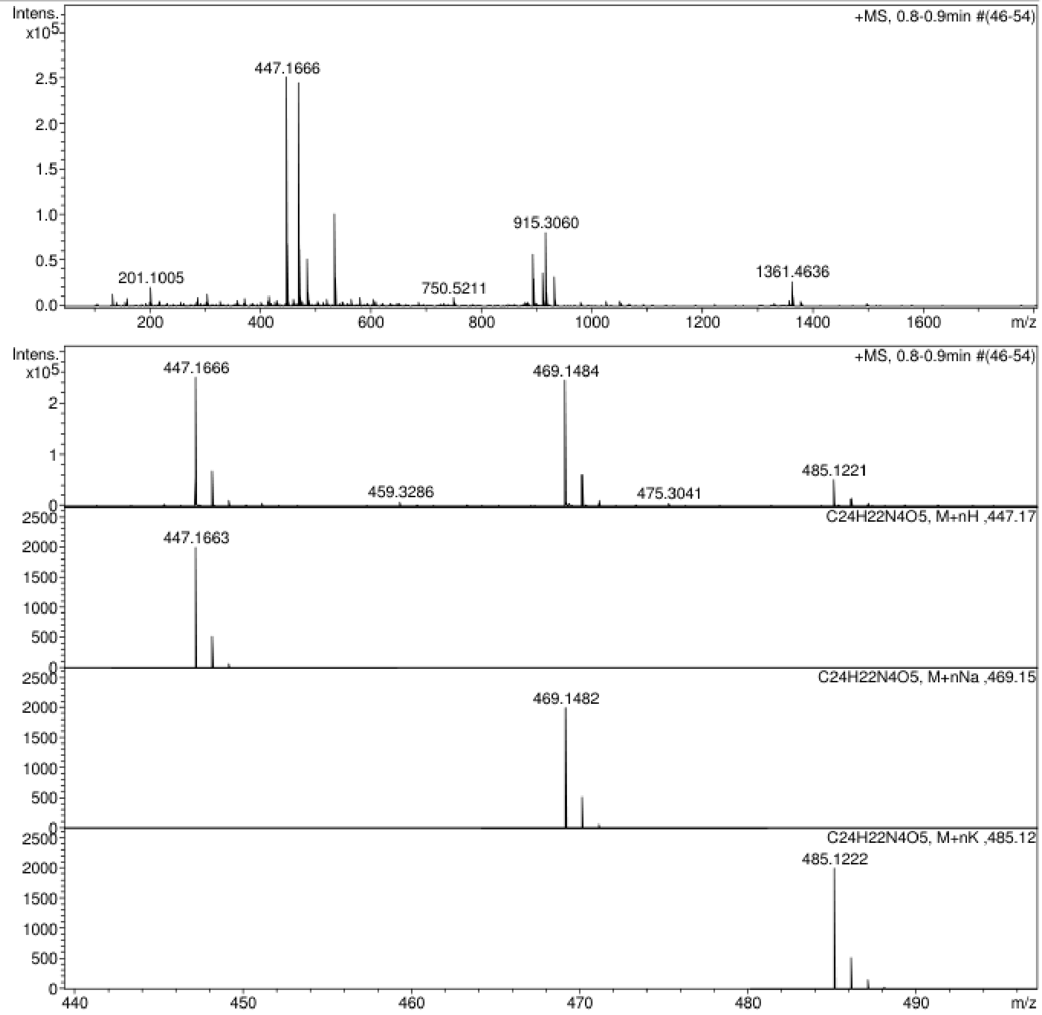


Bruker Compass DataAnalysis 4.0 printed: 20.01.2026 15:36:16 Page 1 of 1

**Figure S18:** HRMS spectrum of compound **2b**

# Display Report

**Analysis Info**

| | | | |
|---|---|---|---|
| Analysis Name | D:\Data\Kolotyrkina\2026\Koloshnikova\0120007.d | Acquisition Date | 20.01.2026 15:38:28 |
| Method | tune_100-1200.m | Operator | BDAL@DE |
| Sample Name | /FEDU VK507 | Instrument / Ser# | maXis 43 |
| Comment | C25H24N4O4 CH3CN | | |

**Acquisition Parameter**

| | | | | | |
|---|---|---|---|---|---|
| Source Type | ESI | Ion Polarity | Positive | Set Nebulizer | 1.0 Bar |
| Focus | Active | | | Set Dry Heater | 200 °C |
| Scan Begin | 50 m/z | Set Capillary | 4500 V | Set Dry Gas | 4.0 l/min |
| Scan End | 1800 m/z | Set End Plate Offset | -500 V | Set Divert Valve | Waste |

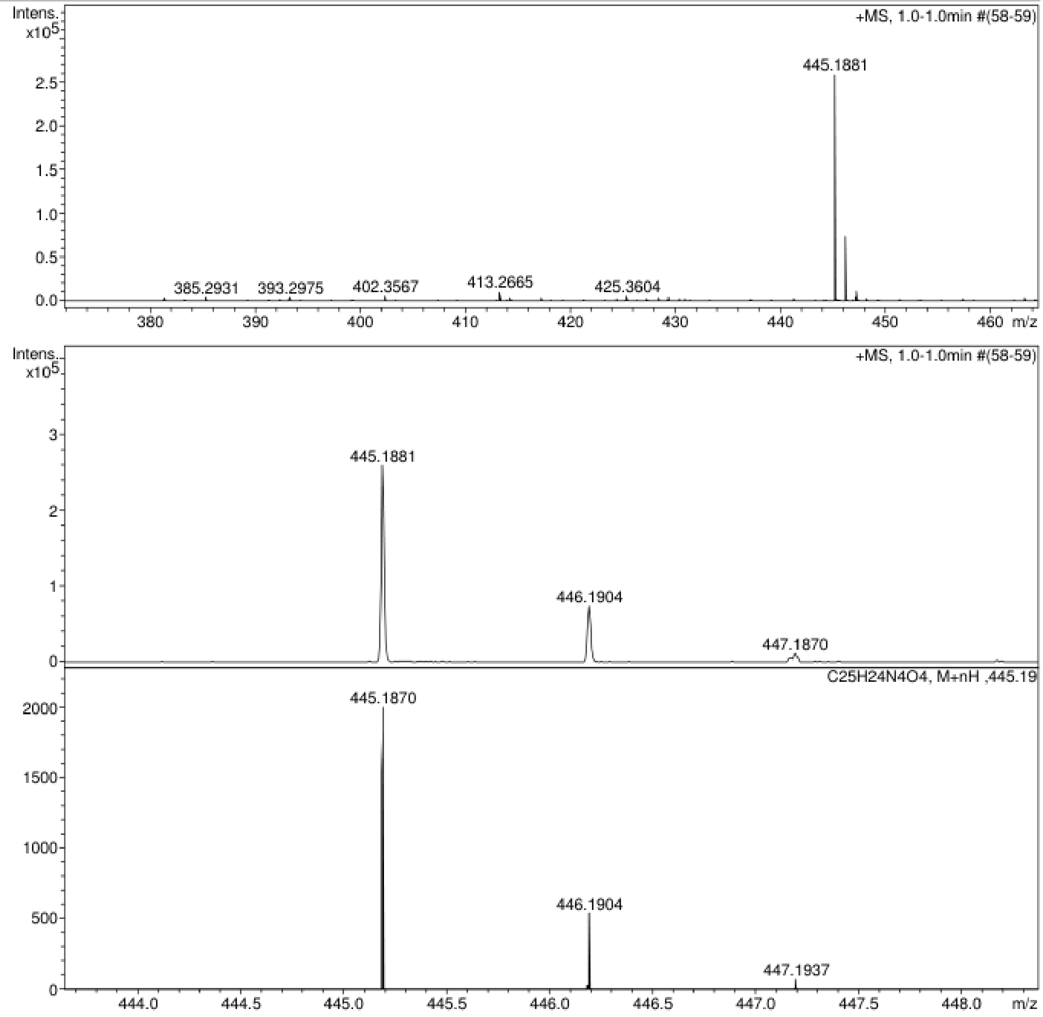


Bruker Compass DataAnalysis 4.0 printed: 20.01.2026 15:42:04 Page 1 of 1

**Figure S19:** HRMS spectrum of compound **2c**

# Display Report

**Analysis Info**

| | | | |
|---|---|---|---|
| Analysis Name | D:\Data\Ilyushenkova\VK60214.d | Acquisition Date | 24.05.2026 13:23:45 |
| Method | tune_100-1200.m | Operator | BDAL@DE |
| Sample Name | /FEDU VK60214 | Instrument / Ser# | maXis 43 |
| Comment | | | |

**Acquisition Parameter**

| | | | | | |
|---|---|---|---|---|---|
| Source Type | ESI | Ion Polarity | Positive | Set Nebulizer | 1.0 Bar |
| Focus | Active | | | Set Dry Heater | 200 °C |
| Scan Begin | 50 m/z | Set Capillary | 4500 V | Set Dry Gas | 4.0 l/min |
| Scan End | 1800 m/z | Set End Plate Offset | -500 V | Set Divert Valve | Waste |

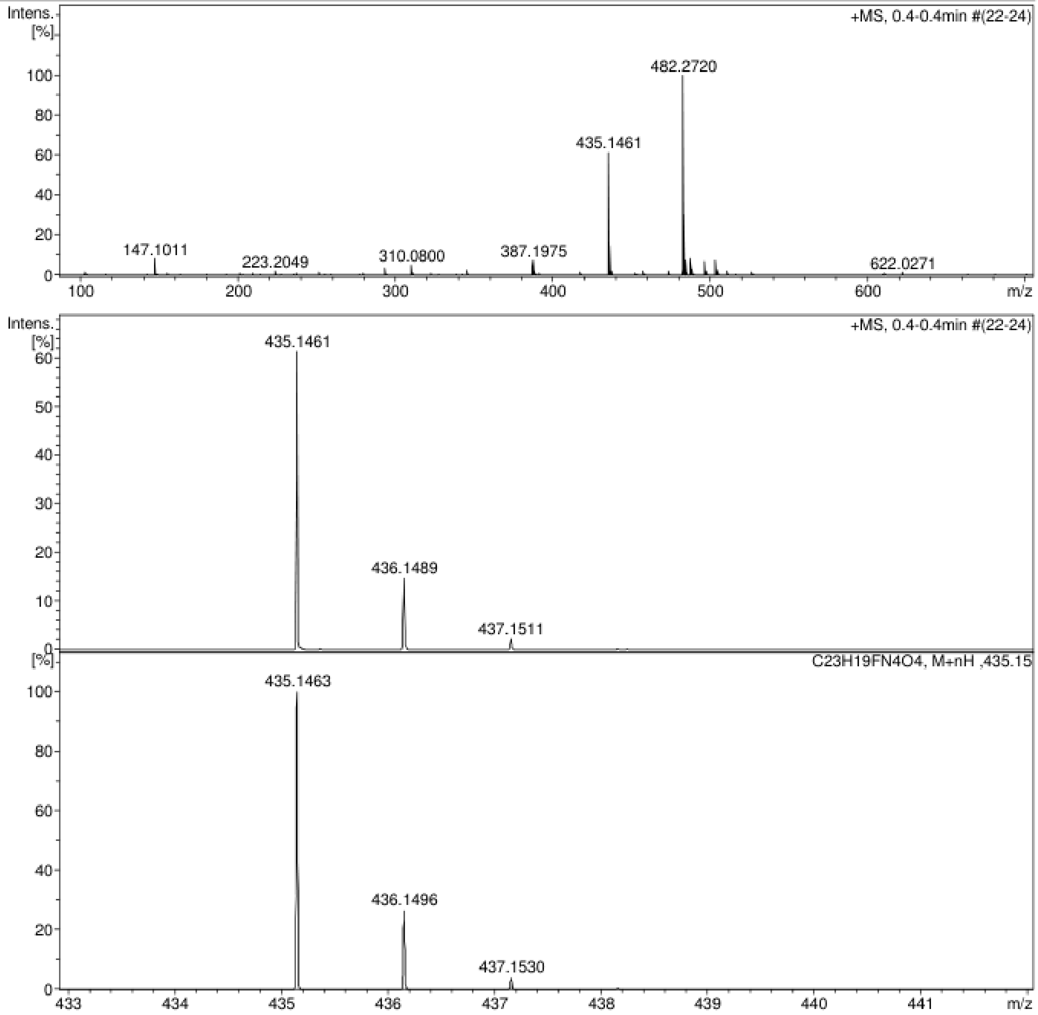


Bruker Compass DataAnalysis 4.0 printed: 24.05.2026 13:25:44 Page 1 of 1

**Figure S20:** HRMS spectrum of compound **2d**

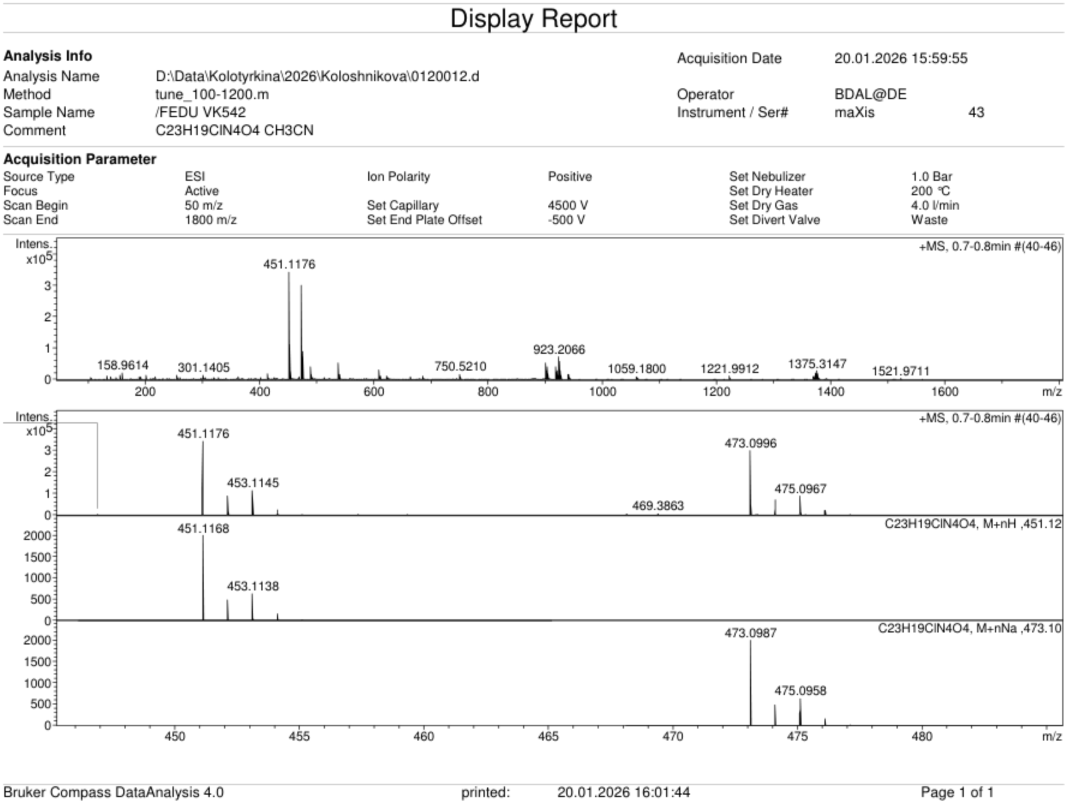


**Figure S21:** HRMS spectrum of compound **2e**

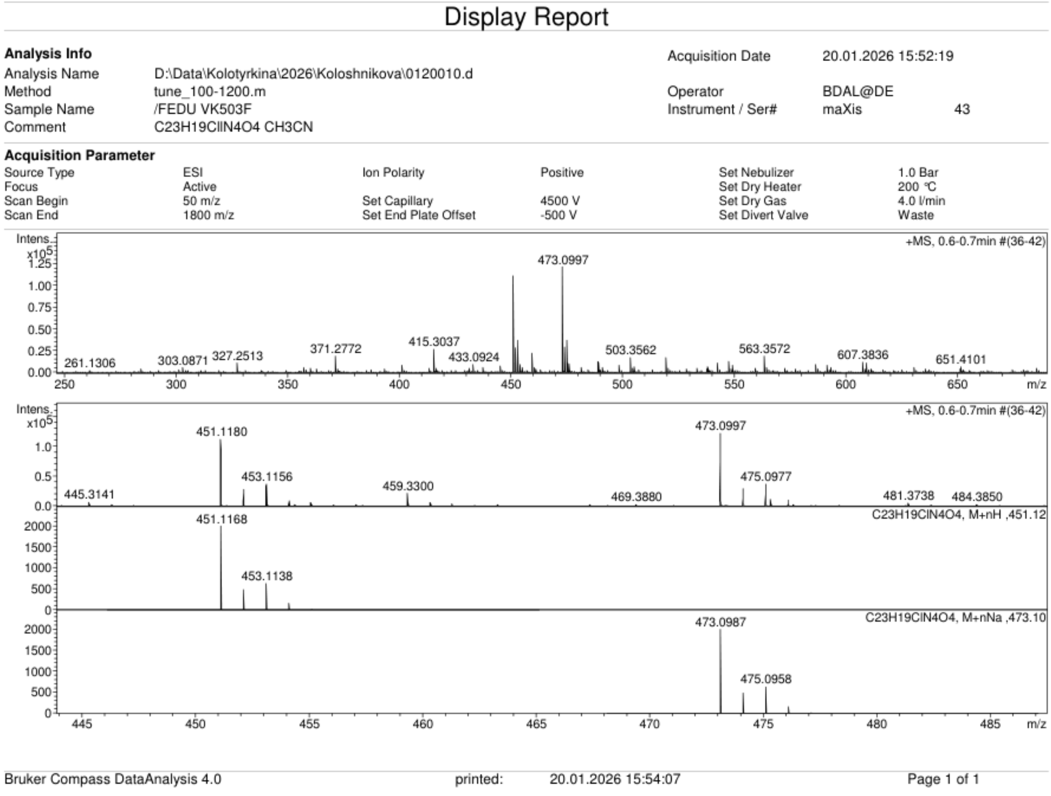


**Figure S22:** HRMS spectrum of compound **2f**

## Display Report

**Analysis Info**

| | | | |
|---|---|---|---|
| Analysis Name | D:\Data\Kolotyrkina\2026\Koloshnikova\0203004.d | Acquisition Date | 03.02.2026 13:03:40 |
| Method | tune_low.m | Operator | BDAL@DE |
| Sample Name | /FEDU VK541M | Instrument / Ser# | micrOTOF 10248 |
| Comment | C23H19BrN4O4 clb added CH3CN | | |

**Acquisition Parameter**

| | | | | | |
|---|---|---|---|---|---|
| Source Type | ESI | Ion Polarity | Positive | Set Nebulizer | 0.4 Bar |
| Focus | Not active | | | Set Dry Heater | 180 °C |
| Scan Begin | 50 m/z | Set Capillary | 4500 V | Set Dry Gas | 4.0 l/min |
| Scan End | 3000 m/z | Set End Plate Offset | -500 V | Set Divert Valve | Waste |

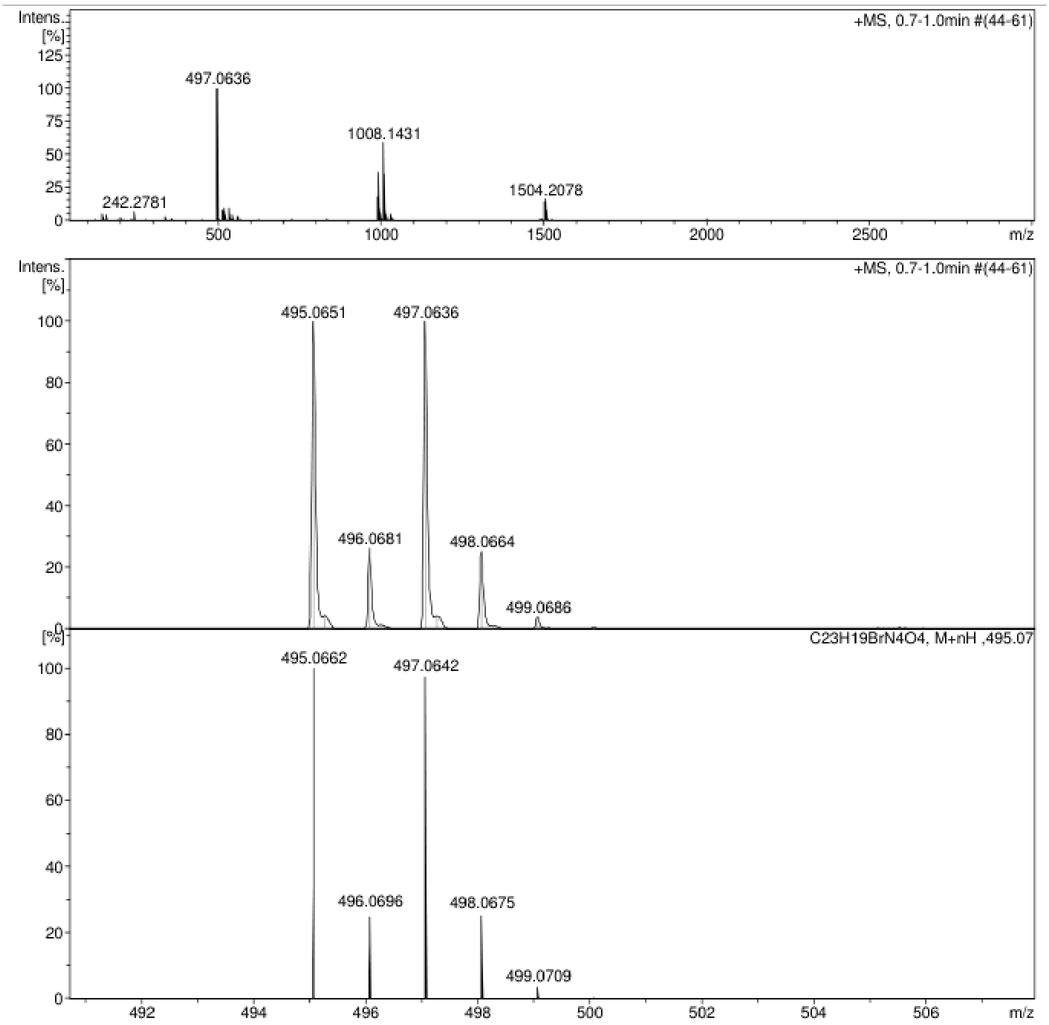


Bruker Compass DataAnalysis 4.0 printed: 03.02.2026 13:07:15 Page 1 of 1

**Figure S23:** HRMS spectrum of compound **2g**

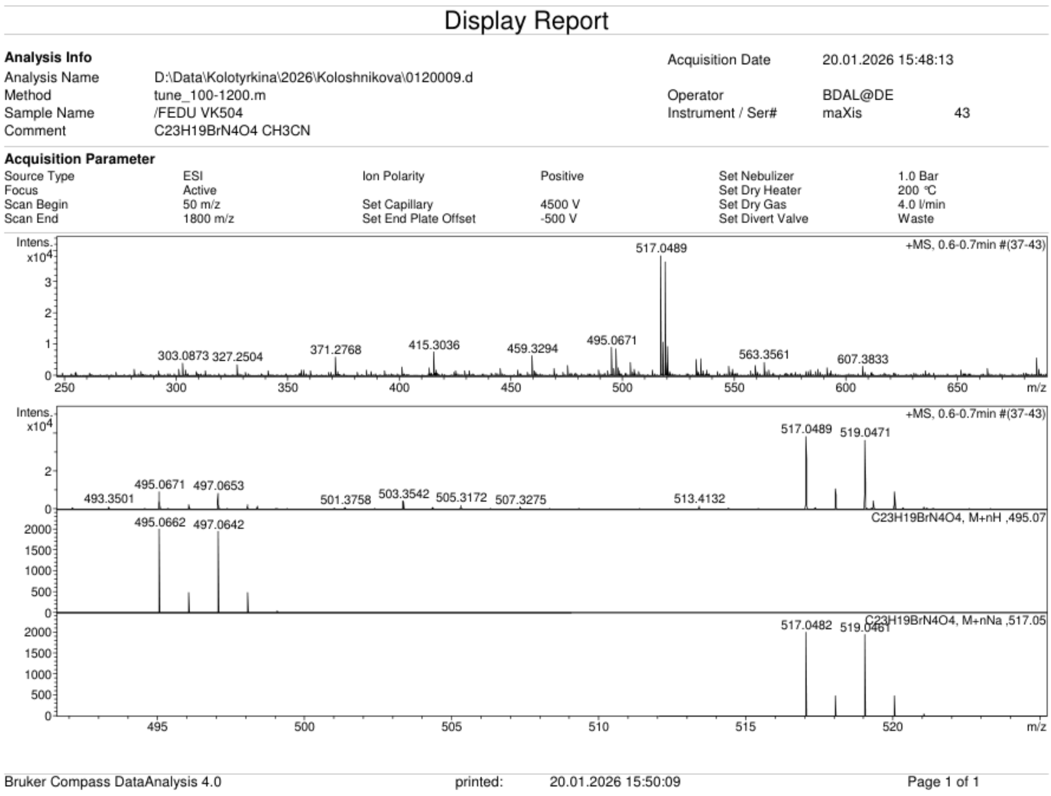


**Figure S24:** HRMS spectrum of compound **2h**
